# Novel Loci for Metabolic Networks and Multi-Tissue Expression Studies Reveal Genes for Atherosclerosis

**DOI:** 10.1371/journal.pgen.1002907

**Published:** 2012-08-16

**Authors:** Michael Inouye, Samuli Ripatti, Johannes Kettunen, Leo-Pekka Lyytikäinen, Niku Oksala, Pirkka-Pekka Laurila, Antti J. Kangas, Pasi Soininen, Markku J. Savolainen, Jorma Viikari, Mika Kähönen, Markus Perola, Veikko Salomaa, Olli Raitakari, Terho Lehtimäki, Marja-Riitta Taskinen, Marjo-Riitta Järvelin, Mika Ala-Korpela, Aarno Palotie, Paul I. W. de Bakker

**Affiliations:** 1 Medical Systems Biology, Departments of Pathology and of Microbiology and Immunology, The University of Melbourne, Parkville, Victoria, Australia; 2 Immunology Division, Walter and Eliza Hall Institute of Medical Research, Parkville, Victoria, Australia; 3 Institute of Molecular Medicine (FIMM), University of Helsinki, Helsinki, Finland; 4 Department of Chronic Disease Prevention, National Institute for Health and Welfare, Helsinki, Finland; 5 Department of Human Genetics, Wellcome Trust Sanger Institute, Wellcome Trust Genome Campus, Hinxton, United Kingdom; 6 Department of Clinical Chemistry, Fimlab Laboratories, Tampere University Hospital and University of Tampere School of Medicine, Tampere, Finland; 7 Division of Vascular Surgery, Department of Surgery, Tampere University Hospital, Tampere, Finland; 8 Department of Medical Genetics, University of Helsinki and Helsinki University Hospital, Helsinki, Finland; 9 Computational Medicine, Institute of Clinical Medicine, Faculty of Medicine, University of Oulu, Oulu, Finland; 10 NMR Metabolomics Laboratory, School of Pharmacy, University of Eastern Finland, Kuopio, Finland; 11 Biocenter Oulu, University of Oulu, Oulu, Finland; 12 Department of Internal Medicine, Clinical Research Center, University of Oulu, Oulu, Finland; 13 Department of Medicine, University of Turku and Turku University Hospital, Turku, Finland; 14 Department of Clinical Physiology, University of Tampere and Tampere University Hospital, Tampere, Finland; 15 Research Centre of Applied and Preventive Cardiovascular Medicine and Department of Clinical Physiology, University of Turku and Turku University Hospital, Turku, Finland; 16 Department of Medicine, University of Helsinki, Helsinki, Finland; 17 Department of Epidemiology and Biostatistics, School of Public Health, Imperial College London, London, United Kingdom; 18 Department of Public Health Science and General Practice, University of Oulu, Oulu, Finland; 19 Program in Medical and Population Genetics, Broad Institute of Harvard and MIT, Cambridge, Massachusetts, United States of America; 20 Division of Genetics, Department of Medicine, Brigham and Women's Hospital, Harvard Medical School, Boston, Massachusetts, United States of America; 21 Department of Medical Genetics, University Medical Center Utrecht, Utrecht, The Netherlands; 22 Department of Epidemiology, University Medical Center Utrecht, Utrecht, The Netherlands; The University of Queensland, Australia

## Abstract

Association testing of multiple correlated phenotypes offers better power than univariate analysis of single traits. We analyzed 6,600 individuals from two population-based cohorts with both genome-wide SNP data and serum metabolomic profiles. From the observed correlation structure of 130 metabolites measured by nuclear magnetic resonance, we identified 11 metabolic networks and performed a multivariate genome-wide association analysis. We identified 34 genomic loci at genome-wide significance, of which 7 are novel. In comparison to univariate tests, multivariate association analysis identified nearly twice as many significant associations in total. Multi-tissue gene expression studies identified variants in our top loci, *SERPINA1* and *AQP9*, as eQTLs and showed that *SERPINA1* and *AQP9* expression in human blood was associated with metabolites from their corresponding metabolic networks. Finally, liver expression of *AQP9* was associated with atherosclerotic lesion area in mice, and in human arterial tissue both *SERPINA1* and *AQP9* were shown to be upregulated (6.3-fold and 4.6-fold, respectively) in atherosclerotic plaques. Our study illustrates the power of multi-phenotype GWAS and highlights candidate genes for atherosclerosis.

## Author Summary

In this study, we aim to identify novel genetic variants for metabolism, characterize their effects on nearby genes, and show that the nearby genes are associated with metabolism and atherosclerosis. To discover new genetic variants, we use an alternative approach to traditional genome-wide association studies: we leverage the information in phenotype covariance to increase our statistical power. We identify variants at seven novel loci and then show that our top signals drive expression of nearby genes *AQP9* and *SERPINA1* in multiple tissues. We demonstrate that *AQP9* and *SERPINA1* gene expression, in turn, is associated with metabolite levels. Finally, we show that the genes are associated with atherosclerosis using mouse atherosclerotic lesion size (*AQP9*) as well as tissue from healthy human arteries and atherosclerotic plaques (*AQP9* and *SERPINA1*). This study illustrates that multivariate analysis of correlated metabolites can boost power for gene discovery substantially. Further functional work will need to be performed to elucidate the biological role of *SERPINA1* and *AQP9* in atherosclerosis.

## Introduction

Five years of genome-wide association studies (GWAS) have successfully identified common variants at >1,000 genomic loci robustly associated with a wide range of human conditions and quantitative traits [1]. Despite this progress, one limitation is that almost all GWAS performed to date have focused on single traits, even in studies involving multiple related phenotypes. Growing evidence for pleiotropy [2], [3], where the same locus is associated with multiple phenotypes, supports the idea that multivariate analysis of multiple phenotypes can provide a substantial boost in power for locus discovery, consistent with simulation studies [4]–[7].

A plethora of metabolites in blood have been described as risk factors for metabolic syndrome, atherosclerosis and coronary artery disease [8], [9]. Using high-throughput nuclear magnetic resonance assays, quantitative profiles of 130 metabolites in two population-based cohorts from Finland, the Cardiovascular Risk in Young Finns Study (YFS) [10] and the Northern Finland Birth Cohort 1966 (NFBC66) [11] have been determined. These metabolites included lipoprotein subclasses of VLDL, LDL, IDL and HDL as well as lipids, amino acids and other small molecules (Materials S1).

**Figure 1:**
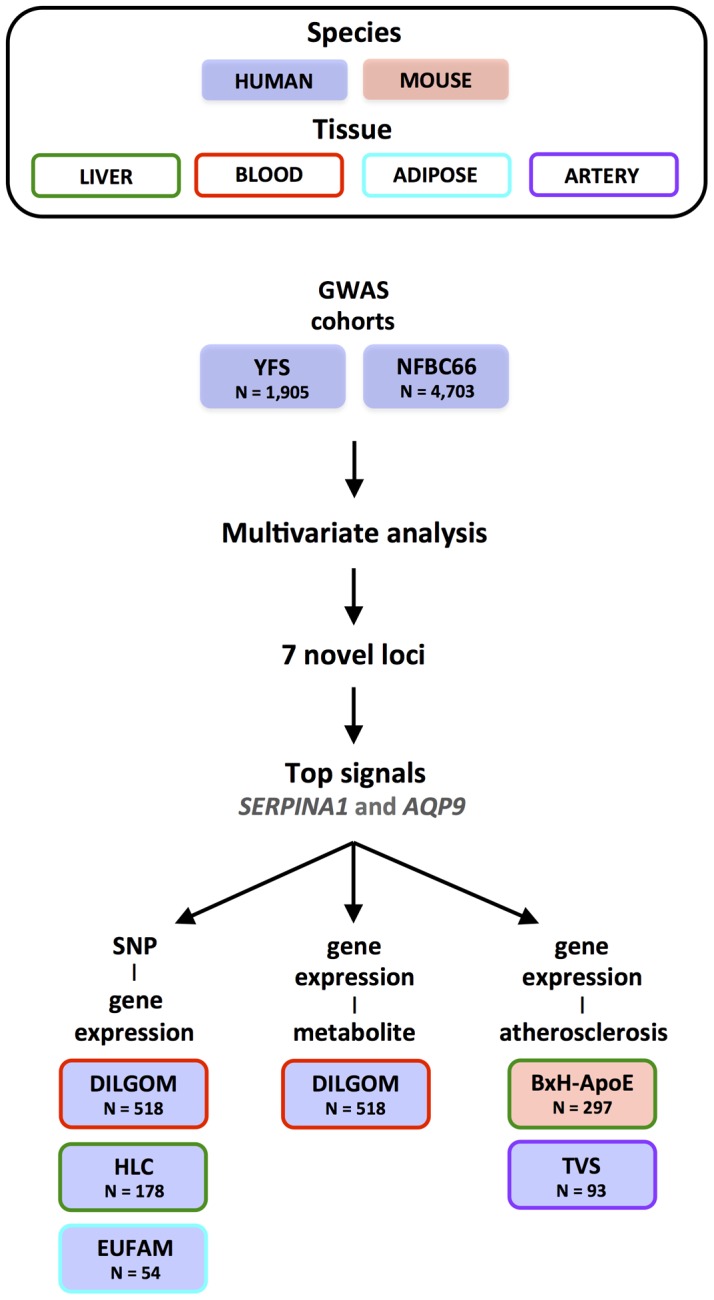
Overview of the study design.

Figure 1 illustrates the general flow of our study. We first applied an unsupervised algorithm to identify networks from the observed correlation structure amongst the 130 metabolite measures in 6,600 individuals. For each of these networks, we performed a multivariate test of association for 2.5 million SNPs [4]. Because we also tested all SNPs for association to each metabolite separately, we can assess the relative gain in power of the multivariate approach. To interpret the novel signals, we tested whether the associated SNPs influenced *cis*-gene expression levels in multiple tissues as well as whether the expression of candidate genes was associated to specific metabolites that drive the initial association. Finally, we analysed arterial tissue from mouse and man to test for a relation between our top candidate genes and atherosclerosis plaques.

## Results

### Genotype and phenotype data

We analysed genotype and phenotype data from the YFS (N = 1,905) [10], [12] and the NFBC66 (N = 4,703) [11], [13]. For both YFS and NFBC66, we imputed SNP genotypes using the MACH algorithm [14] and the HapMap Phase 2 reference panel [15]. Serum collected from both cohorts underwent metabolomic profiling on the same proton nuclear magnetic resonance (NMR) platform [16]. The NMR metabolomics platform used here provided absolute quantitative information on 130 distinct metabolic measures [17]. Metabolite levels for both cohorts were normalized and adjusted for age, gender, cohort, and population structure (Materials and Methods).

### Identification of metabolic networks

**Figure 2:**
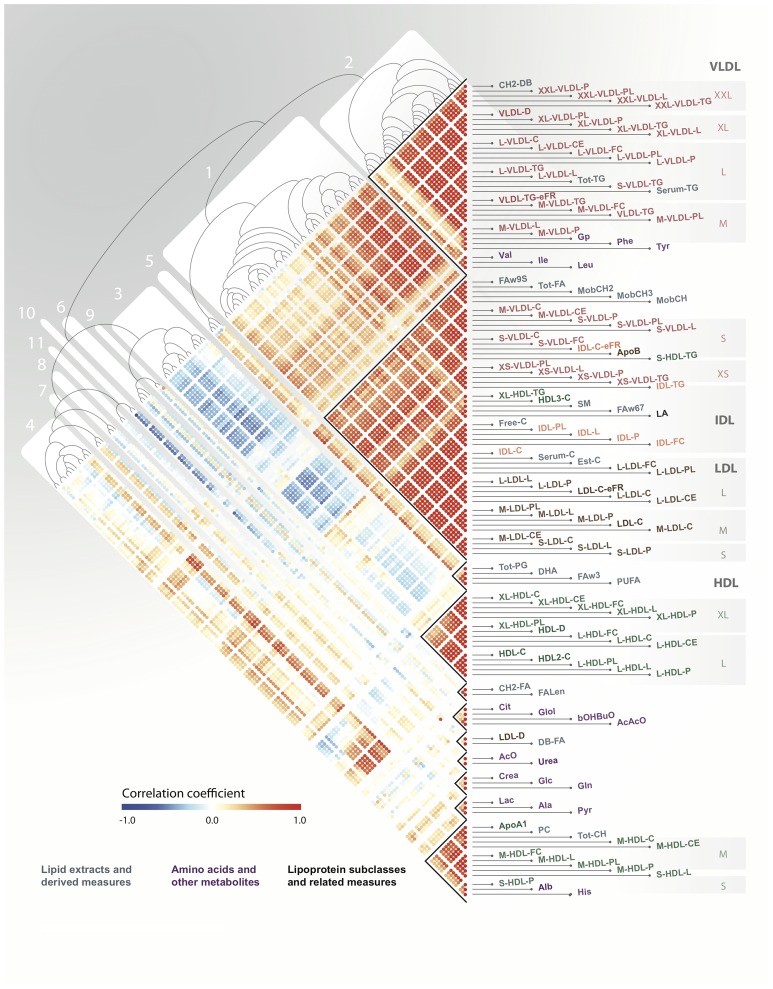
Serum metabolic networks. A Pearson correlation matrix of serum metabolites across both YFS and NFBC66 cohorts was hierarchically clustered and the resulting heatmap and dendrogram are presented here with red indicating high positive correlation, blue high negative correlation, and white no correlation. Clusters of tightly correlated metabolites, metabolic networks, are labeled 1–11.

After correcting for cohort effects and pooling the metabolomic data for YFS and NFBC66, we constructed a Pearson correlation matrix that defined the pairwise relationships between all metabolites and applied agglomerative hierarchical clustering in order to identify networks of metabolites (Figure 2). Using a dynamic, data-driven tree-cutting algorithm [18], we identified 11 metabolic networks that represent various metabolic pathways (Materials and Methods and Figure S1). Additional information for each metabolic network, including full descriptions, abbreviations, inter-metabolite correlations, and supporting association analyses, is given in the Materials S1. Briefly, metabolic network 1 comprises multiple measures related mainly to cholesterol metabolism in the apoB-containing lipoproteins. Metabolic network 2 includes branched-chain and aromatic amino acids together with the large TG-rich VLDL particles and serum triglycerides. Metabolic networks 3 and 4 capture the larger and smaller particles of HDL-metabolism, respectively. Metabolic networks 5, 6, 7, and 8 are related to lipid poly-unsaturation, ketone bodies, the glucose-alanine cycle, and renal function, respectively. Metabolic networks 9, 10, and 11 each contain only 2 metabolites and represent measures of fatty acid chain length and composition, mean diameter of LDL and double-bonding of fatty acid chains, urea and acetate, respectively.

### Genome-wide association analysis

For each of the 11 metabolic networks, we performed SNP association testing using a multivariate test based on Canonical Correlation Analysis and Wilks' lambda [4]. Each association test yielded an *F* statistic, corresponding *P* value, and a loading for each metabolite in the network to indicate the relative contribution of that metabolite to the overall association (Materials and Methods). For univariate analysis, we used standard linear regression where each of the 130 metabolites was regressed onto each SNP.

The implementation of dimensionality reduction and multivariate analysis allowed us to select essentially independent tests based on the correlation structure of the phenotype data. Using multivariate analysis, we tested each SNP only 11 times (one per metabolic network). A Bonferroni correction for testing each SNP to 130 metabolites is overly conservative, since the metabolites are partially correlated, but still common practice [16], [19], [20]. Accordingly, we set genome-wide significance thresholds at *P*<4.5×10^−9^ and *P*<3.8×10^−10^ for multivariate and univariate analysis, respectively.

To maximize power, we performed a joint analysis of both cohorts, correcting for population structure and cohort-specific effects. We observed little evidence for test statistic inflation, lambda range 1.01–1.06 (Materials and Methods and Table S1). Across all 11 metabolic networks, the joint multivariate analysis yielded 713 SNPs significantly associated with one or more metabolic networks (*P*<4.5×10^−9^). This corresponded to 34 distinct loci and 75 significant locus-network associations overall (Table 1 and Materials and Methods). Loci were considered novel if they had not been previously associated at genome-wide significance with a metabolite or other metabolic phenotype in the NHGRI Catalogue of Published GWAS [1] and if they were independent of other proximal signals (Materials and Methods and Table S2). Of the 34 loci detected, 27 were previously identified to be associated with fasting glucose levels [21], [22], total measures of LDL, HDL and triglycerides [23], [24], bradykinin [25], glutamine [16], [25], alanine-valine ratio [16], phenylalanine [16], citrate [16], and sphingolipids [26]. Overall, we found 7 novel loci associated with 12 metabolic networks in total (Table 1).

**Table 1:** Loci detected using joint multivariate association analysis.

Locus	Top SNP	Chr	MAF	Top multivariate Pvalue**	Metabolic network	Top metabolite*	Novel
*PCSK9, USP24*	rs1998013	1p32.3	0.02	4.77E-13	1	IDL-FC#	N
*ANGPTL3, DOCK7*	rs10889332	1p31.3	0.29	8.40E-15	1,2,3,4	MobCH#	N
*GALNT2*	rs10127775	1q42.13	0.44	1.49E-09	1,3	L-HDL-PL	N
*APOB*	rs673548	2p24.1	0.27	9.64E-14	1,2,4	S-VLDL-TG#	N
*GCKR*	rs1260326	2p23.3	0.36	1.31E-12	2,3,4,7	S-HDL-P	N
*SLC1A4*	rs10211524	2p14	0.39	3.13E-10	2	Val	N
*G6PC2*	rs560887	2q24.3	0.31	3.57E-15	8	Glc#	N
*ADAMTS3*	rs12507628	4q13.3	0.18	2.84E-09	4	S-HDL-L	N
*PPM1K, HERC6*	rs1440581	4q22.1	0.47	1.05E-10	2	Val#	N
*CYP4V2, KLKB1*	rs1912826	4q35.2	0.43	3.72E-12	2,4	Phe	N
*PFN3, F12, GRK6*	rs2731672	5q35.3	0.27	3.15E-14	2	Phe	N
*ELOVL2*	rs3798722	6p24.2	0.12	3.65E-09	5	DHA	N
*PPP1R3B, TNKS*	rs4841132	8p23.1	0.15	2.35E-09	4	M-HDL-FC	N
*LPL*	rs12678919	8p21.3	0.09	9.22E-13	1,2,3	M-VLDL-PL#	N
*ABCA1*	rs4149310	9q31.1	0.1	2.31E-10	1,3	XL-HDL-P	N
*FADS1/2/3*	rs102275	11q12	0.43	3.88E-264	1,2,3,4,5,9,10	LA#	N
*APOA1/C3/A4/A5*	rs964184	11q23	0.14	8.44E-20	1,2,3,4	S-VLDL-P#	N
*SPRYD4, GLS2*	rs2657880	12q13.2	0.14	7.08E-30	8	Gln#	N
*LIPC*	rs1532085	15q22.1	0.44	8.69E-104	1,2,3,4,10	XL-HDL-TG#	N
*CETP*	rs173539	16q13	0.28	2.78E-70	1,2,3,4,10	XS-VLDL-L#	N
*TAT*	rs4788815	16q22.3	0.35	4.02E-13	2	Tyr#	N
*HP, HPR, DHX38*	rs217181	16q22.3	0.18	1.47E-36	2,6	Gp#	N
*GLTPD2, TM4SF5, VM01*	rs12051548	17p13.2	0.06	1.08E-11	1	SM	N
*LDLR*	rs6511720	19p13.2	0.1	3.87E-09	4	Tot-CH	N
*APOE/C1/C2*	rs445925	19q13.32	0.06	5.71E-42	1,3,4	L-LDL-FC#	N
*PLTP*	rs4810479	20q13.12	0.27	2.15E-42	1,2,3,4	XL-HDL-TG#	N
*GSC2, SLC25A1, CLTCL1*	rs712964	22q11.21	0.41	2.94E-11	6	Cit#	N
*GC*	rs1851024	4q13.3	0.05	1.07E-14	1,4	Alb	Y
*CXCL5, PF4, PPBP*	rs16850360	4q13.3	0.03	3.40E-10	4	Alb	Y
*EREG*	rs2168889	4q13.3	0.05	5.76E-14	4	Alb	Y
*SERPINA1*	rs1303	14q32.13	0.24	5.42E-48	1,2	IDL-C	Y
*AQP9*	rs16939881	15q22.1	0.05	2.92E-27	1,2,3,4	XL-HDL-TG#	Y
*MYO1E, CCNB2, RNF111*	rs2306786	15q22.2	0.17	9.55E-11	2	Tot-TG	Y
*ZFHX3*	rs10500569	16q22.3	0.23	7.00E-12	2	Tyr	Y

* Complete metabolic network loadings, indicating the relative contributions of single metabolites to the overall association, are given in Figure S4.

** Multiple test corrected significance threshold for a metabolic network association was *P*<4.5×10^−9^.

# Indicates that the top metabolite was also detected by univariate test.

**Figure 3:**
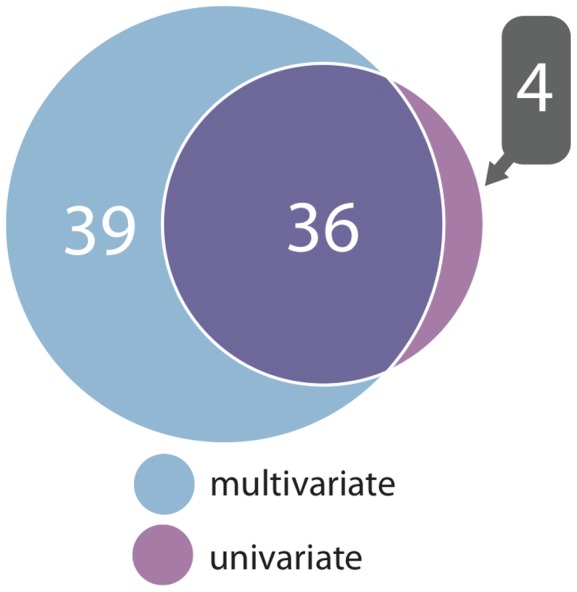
Associations detected between genomic loci and metabolic networks. A Venn diagram showing the number of associations between all genomic loci and metabolic networks stratified by joint multivariate and univariate analysis (for univariate, at least one metabolite from a network need be associated).

In comparing multivariate and univariate *P* values for a given SNP, we selected the lowest univariate *P* value for any single metabolite in a given network. We found that multivariate tests yielded more significant *P* values, reflecting increased power compared to univariate tests (Figure S2). In terms of number of significant associations, multivariate analysis outperformed univariate in both cohorts. When their respective genome-wide significance thresholds are applied, multivariate analysis uncovered 75 locus-metabolic network associations, whereas univariate analysis found only 40 (almost all of which were detected by multivariate analysis), leading to the detection of 7 novel loci instead of one (Figure 3). This demonstrates the relative gain of multivariate testing compared to univariate testing. Notably, multivariate analysis still uncovered more associations (69% more) than univariate analysis even when applying the more stringent genome-wide threshold for 130 independent metabolites. Multivariate also outperformed univariate when assessing only known loci, i.e. those with prior genome-wide significant association to metabolites in the NHGRI Catalogue of Published GWAS [1] (Figure S3).

From the multivariate analysis, our strongest association signal overall was due to a nonsynonymous SNP, rs1303 (Glu400Asp) located in the last exon of *SERPINA1*. This variant was associated with metabolic networks 1 and 2 (*P* = 5.4×10^−48^ and *P* = 7.4×10^−22^, respectively; Figure S4). To explore the extent to which rs1303 perturbs protein structure, we utilized the PolyPhen2 algorithm [27]. PolyPhen2 predicted the Glu to Asp mutation to be benign (naïve Bayes posterior probability = 0.0 and 0.005 for the HumDiv and HumVar training sets, respectively). The next strongest signal overall was an intronic SNP (rs16939881) at the *AQP9* locus, associated with metabolic networks 1, 2, 3, and 4 (*P* = 2.9×10^−27^, *P* = 4.9×10^−15^, *P* = 2.3×10^−18^ and *P* = 2.0×10^−14^, respectively; Figure S4). The metabolic network associations at *AQP9* remained highly significant after conditioning on the previously identified *LIPC* locus, 250 Kb downstream (Table S2). We focus on our two top signals for subsequent in-depth analyses. Because our top signals are within the *SERPINA1* and *AQP9* genes, we assume these to be the most likely candidate genes.

### Fine-mapping and conditional analysis of *SERPINA1* and *AQP9*

Using the 1000 Genomes Phase I integrated variant set and the IMPUTE2 algorithm [28], we generated denser maps of genetic variants for the *SERPINA1* and *AQP9* loci. We then performed a multivariate test for each SNP with metabolic networks 1–4 as well as a conditional analysis to ascertain any independent signals in each region.

After 1000 Genomes imputation, rs1303 remained the top signal at the *SERPINA1* locus for metabolic networks 1 and 2. Conditioning on rs1303 revealed an independent association between another nsSNP, rs28929474, and metabolic networks 1 and 2 (*P* = 1.7×10^−19^ and *P* = 3.7×10^−13^ respectively) (Figure S5). Rs28929474 (Glu366Lys) lies in the last exon of *SERPINA1* and, unlike rs1303, it was predicted by PolyPhen2 to be a probable damaging mutation with a naïve Bayes posterior probability = 1.0 for both HumDiv and HumVar.

Imputation of the *AQP9* locus with the 1000 Genomes panel yielded less confidently inferred genotypes than the HapMap2 panel at the top SNP rs16939881 (posterior probability >0.90 for a genotype call). Consequently we had less power at rs16939881, however it still remained significantly associated with metabolic networks 1, 2, and 3. Even with reduced power, conditional analysis showed that the signal at *AQP9* could be explained by rs16939881 alone (Figure S6).

### Novel variants drive expression of *SERPINA1* and *AQP9* in multiple tissues

We next investigated metabolic network associated variants for eQTL effects on *SERPINA1* and *AQP9*. We used three resources (a) the DILGOM cohort, a Finnish population-based cohort (N = 518) with gene expression data (from whole blood) and serum metabolomic data [29], (b) a subset of the EUFAM study (N = 54) with familial low HDL cholesterol phenotype [30] and subcutaneous adipose tissue gene expression data, and (c) the Human Liver Cohort, a Caucasian cohort (HLC; N = 178) with liver tissue gene expression data [31]–[33]. These three resources also comprise genome-wide SNP data. We summarize the eQTL analyses in Table S3.

**Figure 4:**
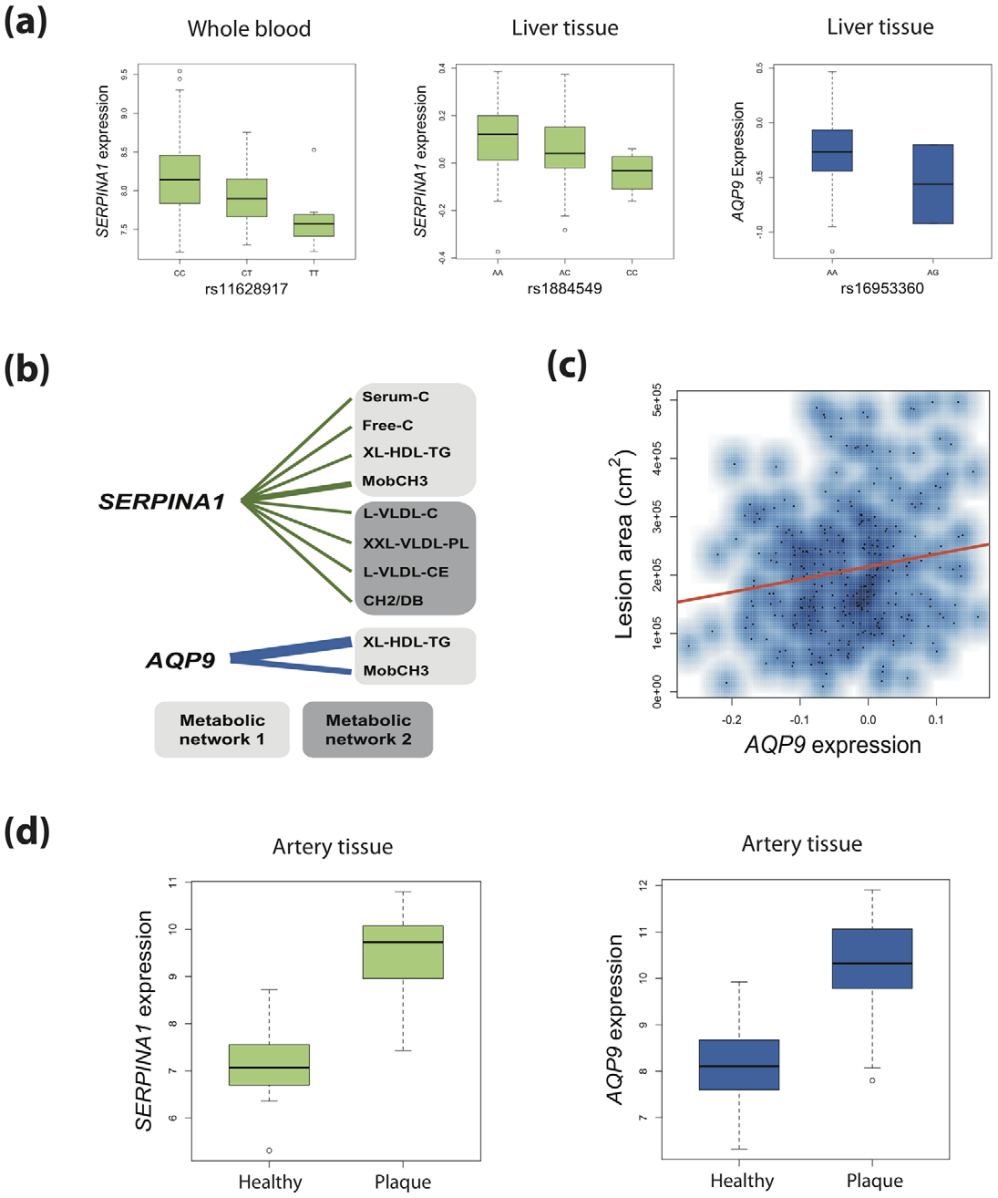
Connecting genetic variation, gene expression, metabolites, and atherosclerosis for *SERPINA1* and *AQP9*. (a) Boxplots show SNPs associated with metabolic networks are also *cis* eQTLs for *SERPINA1* (human blood and liver) and *AQP9* (human liver). Boxplots consist of median log2-normalised expression for each genotype with first and third quartiles designated by box edges. Whiskers extend to +/−1.5 times interquartile range. (b) Human blood expression of *SERPINA1* and *AQP9* was associated with metabolites derived from the same metabolic networks as their corresponding genetic variants. Edge widths are proportional to the strength of association (*P* value). (c) Liver expression of *AQP9* (but not *SERPINA1*) in mice on a hyperlipidemic *APOE* ^−/−^ background showed significant positive association with aortic lesion area. (d) Boxplots for log2-normalised expression of *SERPINA1* and *AQP9* in healthy human arterial tissue versus that for atherosclerotic plaques.

In the DILGOM study, the SNP explaining the most variance in *SERPINA1* expression (rs11628917; linear regression *P* = 6.0×10^−10^; adjusted R^2^ = 0.07) was also strongly associated with metabolic networks 1 and 2 from the YFS and NFBC66 joint analysis (*P* = 9.6×10^−14^ and *P* = 1.9×10^−11^) (Figure 4). In our data, there was moderate linkage disequilibrium (LD; r^2^ = 0.47; D′ = 0.99) between the top *SERPINA1* SNP (rs1303) and the blood eQTL (rs11628917). Conditional analysis showed that the association of rs11628917 with both metabolic networks could be explained by rs1303, suggesting non-independence. Rs1303 itself was nominally associated with *SERPINA1* expression (*P* = 0.01). The independent signal at rs28929474 showed no evidence of influencing *SERPINA1* expression (and was not in LD with any eQTLs), suggesting that its primary effect may be protein structure destabilization. No blood eQTLs were detected for *AQP9*.

In the EUFAM study, the top and bottom 10^th^ percentiles of HDL-C concentrations (Finnish population age and sex specific percentiles) were used to define high and low HDL-C groups (N = 19 and N = 35, respectively). First, we tested for differences in *AQP9* and *SERPINA1* between high and low HDL-C groups. Both *AQP9* and *SERPINA1* expression were upregulated in adipose tissue of individuals with low HDL-C (fold changes 3.47 and 2.29, *P* = 9.0×10^−4^ and *P* = 0.03, respectively). Analysis of genetic variants did not yield any eQTLs at the *AQP9* the locus. Given the independent but proximal signals at *AQP9* and *LIPC*, we did detect an eQTL 210 Kb downstream within the *LIPC* locus that influenced the adipose expression of *AQP9* (rs1825955; *P* = 4.8×10^−3^) but not *LIPC*. There was low LD between rs1825955 and the top multivariate *AQP9* SNP rs16939881 (r^2^ = 0.17; D′ = 0.94). *SERPINA1* also did not harbour adipose eQTLs (including rs11628917, *P*>0.05), indicating either potentially tissue-specific function of the SNP or lack of statistical power.

The HLC allowed for the analysis of gene expression in the human liver. In the HLC, we detected eQTLs for both *SERPINA1* and *AQP9* (Figure 4). An eQTL in the promoter region of *SERPINA1* explained 3.9% of the liver expression of the gene (rs1884549; *P* = 4.3×10^−3^). Rs1884549 was also associated with metabolic network 1 (*P* = 9.6×10^−22^) and in moderate LD (r^2^ = 0.38; D′ = 0.99) with rs1303. A variant within *AQP9* was associated with its expression in the liver (rs16953360; *P* = 4.6×10^−3^; adjusted R^2^ = 0.04) as well as metabolic networks 1–4 (*P* = 1.0×10^−25^; *P* = 4.7×10^−14^; *P* = 8.1×10^−18^; *P* = 7.1×10^−14^ respectively). SNPs rs16953360 and rs16939881 are in very strong LD (r^2^ = 0.97; D′ = 0.98).

### *SERPINA1* and *AQP9* expression is associated with metabolites

We next investigated whether there was a relationship between *SERPINA1* and *AQP9* and metabolites levels in the DILGOM cohort. To do this, we considered those metabolic networks associated with *SERPINA1* and *AQP9* SNPs then regressed individual metabolite levels on gene expression (Table S4). Genetic variation in *AQP9* was associated with metabolic networks 1–4 and here we observed significant association between expression of *AQP9* and two metabolites from network 1 (XL-HDL-TG: *P* = 8.5×10^−9^; MobCH3: *P* = 7.2×10^−5^). *SERPINA1* harboured genetic variants associated with metabolic networks 1 and 2, and expression of *SERPINA1* was associated with eight metabolites, four from metabolic network 1 and four from metabolic network 2 (Table S4).

### *SERPINA1* and *AQP9* expression is associated with atherosclerosis

Since genetic variation and gene expression of *SERPINA1* and *AQP9* were associated with lipoprotein levels, lipid transporters central to atherosclerosis, we investigated the relationship between these genes and atherosclerosis.

We first investigated a mouse model (BxH-ApoE, N = 298) on a hyperlipidemic apolipoprotein-E (*ApoE*) null background with liver gene expression profiles and quantified aortic lesions [34]–[36]. BxH-ApoE consisted of an F2 population derived from a backcross of mice highly susceptible to atherosclerosis (C57BL/6J *ApoE^−/−^*) and highly resistant (C3H/HeJ *ApoE^−/−^*). The F2 population was then fed on a high-fat, western diet for 16 weeks then euthanized at 24 weeks. Using linear regression, we tested for association between liver expression of *Serpina1a* (the mouse ortholog of *SERPINA1*) and *AQP9* and the area of atherosclerotic lesion in the aorta. Expression of *AQP9* showed significant association with atherosclerotic plaque area (*P* = 5.0×10^−3^; Figure 4), with samples in the top decile of *AQP9* expression having on average 29% larger lesion area than those in the bottom decile. The association remained significant after correction for gender, total cholesterol, triglycerides and HDL. On this background, *Serpina1a* expression did not show association with lesion area (*P* = 0.58).

Finally, we utilized the Tampere Vascular Study (TVS) a collection of atherosclerotic plaque samples from patients undergoing peripheral vascular surgery (carotid and femoral endarterectomy and aortic bypass procedures due to atherosclerosis) and control samples from individuals undergoing coronary artery by-pass surgery (Materials and Methods). In TVS, both *SERPINA1* and *AQP9* showed strong association with lesion status (Figure 4). *AQP9* was expressed at a 4.67 fold higher level in lesions compared to controls (Mann Whitney *P* = 4.64×10^−12^), and similarly *SERPINA1* exhibited 6.33 fold higher expression (Mann Whitney *P* = 2.49×10^−13^). The TVS results suggest that both *AQP9* and *SERPINA1* are candidate genes for atherosclerosis.

## Discussion

We have empirically demonstrated the power of multivariate association testing of metabolite networks. We detected 7 novel loci and investigated the gene expression of our top loci, *SERPINA1* and *AQP9*, in multiple human tissues as well as their potential role in atherosclerosis.

*SERPINA1* was associated with metabolic networks 1 and 2 (top metabolites: total cholesterol in IDL and mean diameter of VLDL, respectively), which are mainly related to cholesterol and triglyceride pathways of apoB-containing lipoproteins as well as diabetes associated amino acids [37]. *SERPINA1* encodes alpha 1-antitrypsin (A1AT), a protease inhibitor that protects surrounding tissues at sites of inflammation, and various studies have suggested A1AT's role in atherosclerosis. A1AT has been detected within HDL particles but not LDL [38], although complexes of A1AT and LDL have been found in the intimal arterial wall and in human atherosclerotic lesions in the coronary artery [39]. Proteolytic degradation of LDL by murine peritoneal macrophages has been shown to be enhanced by A1AT binding, and immunostaining and *in situ* hybridization have also suggested that A1AT is produced by macrophages in the arterial wall [39].

*AQP9* encodes aquaporin 9, a liver glycerol channel [40], and contains variants which showed association with metabolic networks 1 and 2 (top metabolites: triglycerides in very large HDL and mean diameter of VLDL) as well as networks 3 and 4 (top metabolites: mean diameter of HDL and phosphatidylcholine). The proximity of *AQP9* to the well-known *LIPC* gene 250 Kb downstream raises the question of whether the *AQP9* and *LIPC* loci harbour independent effects. Our conditional analyses of metabolic network associated SNPs indicate that these are indeed independent genetic signals. In addition, *LIPC* expression in whole blood from DILGOM was not associated with metabolites from the relevant networks 1, 2, 3, or 4, and *LIPC* liver expression in mouse only slightly attenuated the association of *AQP9* with atherosclerotic lesion area in a linear model (*P* = 0.059). In human aorta, *LIPC* was nominally differentially expressed between healthy and plaque samples (*P* = 0.01) and did not affect the substantially larger aortic differential expression of *AQP9*. Previous experiments have shown AQP9's involvement in gluconeogenesis. AQP9 mRNA and protein have been shown to be greater in human obese T2D patients relative to lean normoglycemics in adipose tissue [41]. The opposite is true in liver, suggesting that reduction in glycerol influx in hepatocytes via AQP9 could prevent excessive lipid accumulation and may reduce hyperglycaemia in obesity [41]. Further, *AQP9^−/−^* mice have previously been shown to have elevated levels of plasma glycerol and triglycerides, and inhibition of AQP9 by a small molecule inhibitor showed that it is required for glycerol-dependent glucose production in murine hepatocytes [42], [43].

Our findings for *SERPINA1* and *AQP9* are consistent with the above studies suggesting associations with cardiometabolic risk factors and show that (a) common variants in both are associated with metabolic networks, (b) these variants modulate gene expression and suggest that there may be potential heterogeneous genetic control in different tissues, (c) expression of both genes was associated with metabolites from the relevant networks, and finally (d) gene expression was positively associated with atherosclerotic lesion area in mice (*AQP9*) and upregulated in atherosclerotic tissue in humans (*SERPINA1* and *AQP9*). We also speculate that the roles of *SERPINA1* and *AQP9* in atherosclerosis are tissue-specific where *AQP9* displays an effect in both liver and arterial tissue and *SERPINA1* only in the latter.

Of the five other novel loci, there were variants proximal to *ZFHX3* on Chr 16, *MYO1E* on Chr 15, as well as three independent signals at 4q13. *ZFHX3* encodes ATBF1, a transcription factor involved in neuronal differentiation and survival [44], [45] that has also been previously implicated in Kawasaki disease, atrial fibrillation and ischemic stroke [46], [47]. Variants at the *ZFHX3* locus were associated with metabolic network 2 where the metabolite with the greatest loading was tyrosine. Little is known about the role of the tyrosine in circulation, however a recent study [37] investigating the predictive ability of five amino acids for type 2 diabetes onset suggested that amino acid metabolism, including tyrosine, plays a role in the pathophysiology of metabolic syndrome, where it is known that individuals with either metabolic syndrome and/or diabetes are at increased risk for stroke. At 4q13, a band that contains the *ALB* albumin gene, metabolic networks 1 and 4 were associated with variants 30 Kb upstream of group-specific component, a vitamin D binding protein (top metabolites: triglycerides in IDL and albumin, respectively). Metabolic network 4, with albumin as the top metabolite, was also associated with variants 10 Kb upstream of *EREG* and independent variants 8 Kb upstream of *CXCL5*. *EREG* encodes epiregulin, part of a family of epidermal growth factors for which there is evidence that osmotic pressure has a role in signal transduction [48], and *CXCL5* encodes a cytokine that has previously been linked with obesity and insulin resistance [49]. Finally, 15q22 harboured intronic variants within the *MYO1E* gene, a non-muscle class I myosin protein, associated with metabolic network 2 (top metabolite: total triglycerides). Myosin 1E has previously been shown to bind phospholipids [50], regulate podocyte function and glomerular filtration [51], as well as contain nsSNPs which display linkage to kidney disease [52].

This study illustrates the importance of accounting for fine-scale phenotypic structure. Although the current GWAS paradigm is based on the testing of one phenotype and one marker at a time, the quantitative phenotype profiles of individuals and corresponding biological samples are rapidly expanding in scope and depth. We are being faced with more complex multivariate phenotypic information, and biologically heterogeneous phenotypes can now be fine-mapped to reveal more informative patterns of association. Powerful statistical approaches that leverage the network covariance can provide novel insights and link genetics with disease.

## Materials and Methods

### Cohorts

The Cardiovascular Risk in Young Finns Study (YFS) is a population based prospective cohort study. It was conducted at 5 university departments of medical schools in Finland (i.e. Turku, Helsinki, Kuopio, Tampere and Oulu), with the aim of studying the levels of cardiovascular risk factors in children and adolescents in different parts of the country. The latest follow-up was conducted in 2007. The serum samples for this metabolomics study were collected at the latest follow up. The study and data collection protocols have been described in detail in [10]. The YFS study protocols have been approved by local ethics committees.

The Northern Finland Birth Cohort 1966 (NFBC66) has been described in detail previously [11]. The original study design focused to study factors affecting pre-term birth, low birth weight, and subsequent morbidity and mortality. Mothers living in the two northern-most provinces of Finland were invited to participate if they had expected delivery dates during 1966. Individuals still living in the Helsinki area or Northern Finland (N = 4,703) were asked to participate in a detailed biological and medical examination as well as a questionnaire at the age of 31 years. The NFBC66 study protocols have been approved by local ethics committees.

The subjects used in the adipose tissue eQTL analysis were obtained from the EUFAM study (European Multicenter Study of Familial Dyslipidemias) database [30] including a Finnish cohort with familial low HDL-C phenotype. The Ethical Committee of the Department of Medicine, Helsinki University Central Hospital approved the EUFAM study. Top and bottom 10^th^ percentiles of HDL-C concentrations (Finnish population age and sex specific percentiles) were used to define the high and low HDL-C groups, respectively, and subject who were not matched for BMI were removed. Subcutaneous adipose tissue biopsies were obtained from 54 individuals. Out of these, 35 individuals had low HDL-C and 19 individuals high HDL-C. Individuals in both low and high HDL-C groups were matched by age and gender. Fat biopsies were collected, RNA extracted and quantified as previously described [53]. RNA labeling, array processing and scanning were done according to the standard protocol by Affymetrix using HG-U133 (Plus 2.0) arrays. Pre-processing of the expression data was done using GC-RMA normalization. Genotyping was performed using the HumanCNV370v1_C platform at the Broad Institute. SNPs with genotype rate <90% were excluded from the analyses and samples were removed if fewer than 95% of SNPs could be genotyped in them.

In the Tampere vascular study (TVS), vascular samples were collected from patients undergoing peripheral vascular surgery due to symptomatic atherosclerosis (cerebrovascular disease due to carotid stenosis, peripheral arterial disease). All of these patients had a polyvascular disease which had affected at least two different vascular beds. Control samples were taken from left internal thoracic arteries (LITA) during coronary artery by-pass surgery (n = 25). Atherosclerotic plaques were collected by endarterectomy from the following arterial sites: femoral artery (n = 24) carotid artery (n = 29) and abdominal aorta (n = 15) all together from a total of 93 patients. The vascular samples were classified according to the American Heart Association classification (AHA) [54]. The carotid and femoral artery samples were of type V or VI, aortic samples were type VI and all control vessels were macroscopically and microscopically healthy. The samples were taken from patients subjected to open vascular surgical procedures at the Division of Vascular Surgery, Tampere University Hospital. The study was approved by the Ethics Committee of Tampere University Hospital. All patients gave informed consent.

The HLC and BxH-ApoE data was obtained from the Sage BioNetworks repository. A detailed description of the HLC data can be found here [31], [32]. Detailed information on mouse experiments and sample handling can be obtained here [34], [35]. An outlier with extreme lesion area (*Z*-score = 4.166, *P* = 1.5×10^−5^) was removed from analysis. Inclusion of the outlier did not affect significance.

### NMR metabolomics

The samples from the NFBC66, YFS and DILGOM cohorts were analyzed using the same high-throughput serum NMR metabolomics platform [17] providing information on lipoprotein subclass distribution and lipoprotein particle concentrations, low-molecular-weight metabolites such as amino acids, 3-hydroxybutyrate, and creatinine, and detailed molecular information on serum lipids including free and esterified cholesterol, sphingomyelin, saturation and ω-3 fatty acids. Further details of the NMR spectroscopy, data analyses as well as the full metabolite identifications have been described previously [17], [29].

### Metabolomic data processing

Individuals known to be on lipid-lowering therapy or pregnant were excluded from analysis. To calculate residuals for all metabolites, each study included the following as covariates: gender, age (only YFS, the NFBC66 is a birth cohort), and loadings of the first 10 principal components from genetic data to correct for cryptic population stratification. Residuals were normalized using an inverse normal transformation to have a mean of zero and a standard deviation of 1. In combining the metabolomic data for the YFS and NFBC66, residuals for all metabolites also included the cohort as a covariate. Processing of metabolites from the DILGOM cohort has been described previously [29].

### Genotyping and imputation

The YFS and NFBC66 cohorts studied were genotyped using standard protocols on the Illumina 670 BeadArray and Illumina 370CNVduo (Illumina, Inc. San Diego, CA, USA) respectively. Prior to imputation, stringent quality filtering was employed for each cohort. Quality control was performed independently for each study prior to imputation. Low quality SNPs (>5% missingness) and poor DNA samples (>5% individual missingness) were removed. In addition, individuals with high genomic heterozygosity (indicative of sample contamination), gender discrepancies or closely related individuals were removed from the data.

Genotype imputation was performed using the MACH algorithm [14] and the CEPH reference panel from HapMapII [15]. After filtering, sample numbers were 1,905 and 4,703 for the YFS and NFBC66 cohorts respectively. After imputation, SNPs were filtered within each cohort via the estimated squared correlation between imputed and true genotypes (Rsq<0.30), estimated minor allele frequency (MAF<0.01), and Hardy-Weinberg equilibrium exact test (*P*<1.0×10^−6^). After SNP filtering, 2,406,682 and 2,360,512 SNPs in the YFS and NFBC66 cohorts respectively were taken forward for further genome-wide analyses. The intensity cluster plots for the top, directly-genotyped SNPs were visually inspected for failures in genotype assay and calling.

### Metabolite clustering

In order to define matrices of related endogenous variates, groupings of metabolites must be defined. Normalized metabolite measurements across the YFS and NFBC66 cohorts were pooled, and the metabolite-metabolite Pearson correlation matrix was hierarchically clustered. From the resulting dendrogram, metabolite cluster detection was done using a dynamic tree cutting algorithm [18] with a minimum cluster membership of one metabolite. We selected the dynamic tree cutting algorithm because it has been shown to outperform other popular methods in simulations as well as give biologically relevant results on real data [29], [55], [56]. In order to maximize power to detect associations, Ferreira and Purcell showed that, within a phenotype set, one should maximize both the number of phenotypes and the level of correlation between phenotypes [4], however in practice these two parameters are inversely related. That is, given a of phenotype measures and individuals, increasing the number of phenotype clusters leads to increasing correlation within clusters and vice versa. For the dynamic tree cut algorithm, we investigated the sensitivity of cluster splitting using the *deepSplit* parameter. Lower integers values of *deepSplit* correspond to lower sensitivity for cluster splitting and thus fewer clusters. Both high and low sensitivity (*deepSplit* = 4 and *deepSplit* = 0, respectively) for cluster splitting were explored using the YFS discovery cohort. The high setting assigned 11 metabolic networks (Figure S1) whereas the low setting assigned 5 metabolic networks (Figure S7). Both clusterings were empirically assessed using the multivariate test above, and both settings detected the same number of loci at genome-wide significance. This was consistent with the inverse relationship between intra-cluster correlation and number of metabolites per cluster. Given no difference in locus detection, we considered the biological interpretation of the clusters. We noted that the low setting could not differentiate TG-rich VLDL particles nor lipid poly-unsaturation and conflated various energy metabolites with small HDL metabolism. Since it presented more straight-forward biological interpretation, we proceeded to downstream analysis with the high sensitivity, corresponding to 11 metabolic networks.

### Statistical analysis

Association testing of SNPs and metabolites was done using two strategies: univariate linear regression and multivariate Canonical Correlation Analysis (CCA). For the former, we used the standard framework 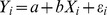 where *Y_i_* is the normalized metabolite measure for individual *i*, *X_i_* is the genotype of the individual at a given SNP (encoded as 0, 1, or 2 for the number of minor alleles), and ε_i_ is a normally distributed random variable with mean equal to zero and constant variance. To implement linear regression, we used the PLINK analysis software [57]. The reported *P* values assume a NULL hypothesis of no association, *b* = 0.

When testing hypotheses that include multiple endogenous variables, the relationships among the endogenous variables must be taken into account in addition to those between the endogenous and exogenous variables. Given these two sets of variables, the aim is to simultaneously find the best predictor of the linear functions of one set as well as the linear function of the other set it best predicts. This yields a pair of variates which are referred to as the first pair of canonical variates. Using the residuals of these linear functions, the process can be repeated to obtain the second pair of canonical variates, and so on. The full sequence of these pairs of variates and their correlations then fully describe the invariant relationships between the endogenous variable set and the exogenous variable set [58].

For multivariate testing, we use the CCA framework implemented in the PLINK.multivariate analysis tool [4] and the R statistical programming language. In this case, the exogenous set consists of only one variable, the SNP, and consequently only one pair of canonical variates is calculated.

Wilks' lambda (λ) is a multivariate analogy of the *F*-test in one-way analysis of variance. In a genetic setting, λ is a statistic which tests for differences between the means of the three genotype groups (AA, AB, and BB) on a combination of endogenous variates (a network of metabolite variables). In this case, λ = 1−ρ^2^ where ρ is the canonical correlation coefficient between the SNP and the network of metabolite variables. The calculation of a *P*-value arises from a transformation of Wilks' lambda into a statistic which is approximately *F* distributed.

Genomic inflation of test statistics can be an indicator of subtle biases in the data and testing (e.g. cryptic population structure). To assess genomic inflation, we compared our observed distribution of −log10(*P*) values to that expected in the absence of association. A linear model was then fitted to the lowest 90% of the distribution and genomic inflation was taken as the slope of the fitted line. Table S1 gives genomic inflation values for multivariate testing of YFS, NFBC66 and meta-analysis across all metabolic networks.

A locus was defined as a 200 kb genomic region centered on the top significantly associated SNP. To determine the independence of the locus-network signals, conditional multivariate association analysis was used for signals either at 4q13.3, close to *LIPC* (i.e. *AQP9* variants), or close to *FADS1/2/3* (i.e. *CD5/CD6* and *INCENP/FTH1/BEST1* variants). For the top SNP(s) at a proximal locus (Table S2), each metabolite in a network was regressed onto the proximal SNP(s) and the resulting residuals were used as endogenous variables in the multivariate test of the target locus. An attenuated signal indicates non-independence, e.g. the SNPs tag the same causal variant. As a result, two loci (*CD5/CD6* and *INCENP/FTH1/BEST1*) were largely attenuated and not regarded as independent from the *FADS1/2/3* locus.

Due to the different number of statistical tests, genome-wide significance differs between multivariate and univariate testing. Here, as the basis of genome-wide significance, we use the common threshold of 5.0×10^−8^, derived from the number of independent common haploblocks in genomes of European descent [59]. Univariate testing of all 130 metabolites, implies a Bonferroni corrected significance level of 5.0×10^−8^/130 = 3.8×10^−10^. Multivariate testing of the metabolic networks we identify here (N = 11) gives a Bonferroni corrected significance level of 5.0×10^−8^/11 = 4.5×10^−9^.

Analysis of the DILGOM cohort considered the bead-weighted and quantile normalized gene expression data from the Illumina HT-12 expression array as described previously [55] and metabolomic measures also described previously [29]. Only those metabolites which were part of the original metabolic network associated with a particular locus were considered. For example, because genetic variation at *SERPINA1* was associated with metabolic networks 1 and 2, expression of *SERPINA1* was only tested for association with metabolites from networks 1 and 2. Since loci were associated with different metabolic networks, different numbers of tests were performed for each candidate gene. We therefore implemented appropriate multiple testing thresholds for each gene where significance was set at *P*<(0.05/total_number_metabolites_in_tested_networks).

For the EUFAM study, statistical eQTL analyses were performed using a linear regression model adjusting for the gender, BMI, and low/high HDL-C affection status. Probe intensities were treated as dependent and genotypes as independent variables. The comparison of gene-expression between the low and high HDL-C groups was performed using a linear regression model. Fold change for each probe was calculated by dividing the mean probe intensity in the low HDL-C group by the mean probe intensity in the high HDL-C group.

For the TVS study, all vascular specimens were immediately frozen and RNA was extracted as previously described [60]. RNA was reverse transcribed into cRNA, biotin-UTP labelled using the Illumina TotalPrep RNA Amplification Kit (Ambion) and cRNA hybridized to the Illumina HumanHT-12 v3 Expression BeadChip. BeadChips were scanned with the Illumina iScan system. Data processing was conducted using R language and appropriate Bioconductor modules. Robust multi-array averaging (RMA) [61] was used to correct negative intensity values after background subtraction. Between arrays normalization was done using robust spline normalization (RSN) [61]. Quality control was performed using sample clustering and multi-dimensional scaling. Seven outliers were removed due to low expression profiles, 4 from carotid artery group and 3 from LITA group.

Fold changes (FCs) for differentially expressed genes were calculated from log2-transformed median expression values between case (carotid, abdominal, femoral) and control group (LITA), and the significance of the differences were evaluated with non-parametric Mann-Whitney U test due to non-normal distribution of expression values and relatively small sample sizes of TVS. If there were more than one probe presenting a gene in the expression chip, the probe with highest median expression value was selected for FC calculation.

## Supporting Information

Figure S1 Hierarchical clustering and detection of 11 metabolite networks.
(TIFF)

Figure S2 Comparison of *P* values from association testing of metabolic networks versus single metabolites. Testing multiple metabolites simultaneously shows an enrichment of low multivariate *P* values. Multivariate and univariate *P* values were compared across all 11 metabolic networks for the 2,406,682 SNPs in the YFS cohort. The different multiple testing burden are shown by dotted lines: horizontal red for multivariate and vertical blue for univariate testing. The univariate *P* value for a SNP was determined via the minimum after testing all single metabolites in a network.
(TIFF)

Figure S3 Associations detected between metabolic networks and loci previously associated with metabolism.
(TIFF)

Figure S4 Loadings from multivariate metabolic network tests.
(TIFF)

Figure S5 Conditional analysis of the *SERPINA1* loci.
(TIFF)

Figure S6 Conditional analysis of the *AQP9* loci.
(TIFF)

Figure S7 Hierarchical clustering and detection of 5 metabolite networks.
(TIFF)

Materials S1 Section describing metabolic networks. Full descriptions, abbreviations, inter-metabolite correlations, and supporting association analyses.
(PDF)

Table S1 Multivariate testing displays little evidence of test statistic inflation.
(XLSX)

Table S2 Conditional association analysis for selected loci. For each network, the metabolite levels were adjusted by regressing out the effects of the SNPs in the ‘Conditional Locus’ column. The corrected metabolite levels in each network were then retested against the target SNP. The multivariate *P* values using both uncorrected metabolite levels (‘Unconditional Pvalue’) and corrected metabolite levels (‘Conditional Pvalue’) are given.
(XLSX)

Table S3 Expression quantitative trait loci across tissues for *AQP9* and *SERPINA1*.
(XLSX)

Table S4 Expression of novel candidate genes is associated with metabolite levels.
(XLSX)
