# Supplementary figures and images for: Novel Loci for Metabolic Networks and Multi-Tissue Expression Studies Reveal Genes for Atherosclerosis
Source: PLoS Genet. 2012 Aug 16;8(8):e1002907. doi: 10.1371/journal.pgen.1002907 (PMC3420921; doi:10.1371/journal.pgen.1002907)

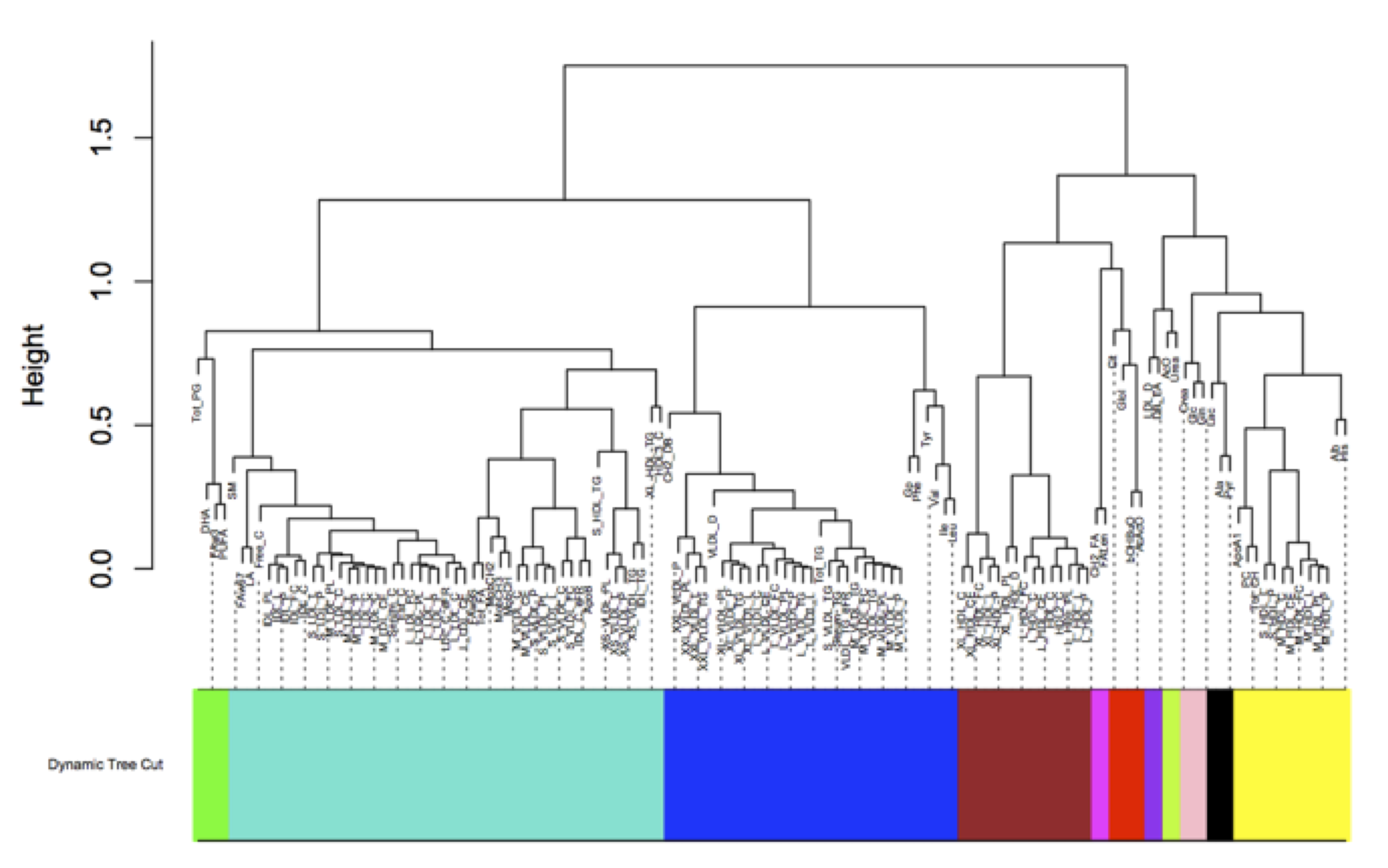

Supplement: Figure S1 — Hierarchical clustering and detection of 11 metabolite networks. (TIFF) [file pgen.1002907.s001.tiff]

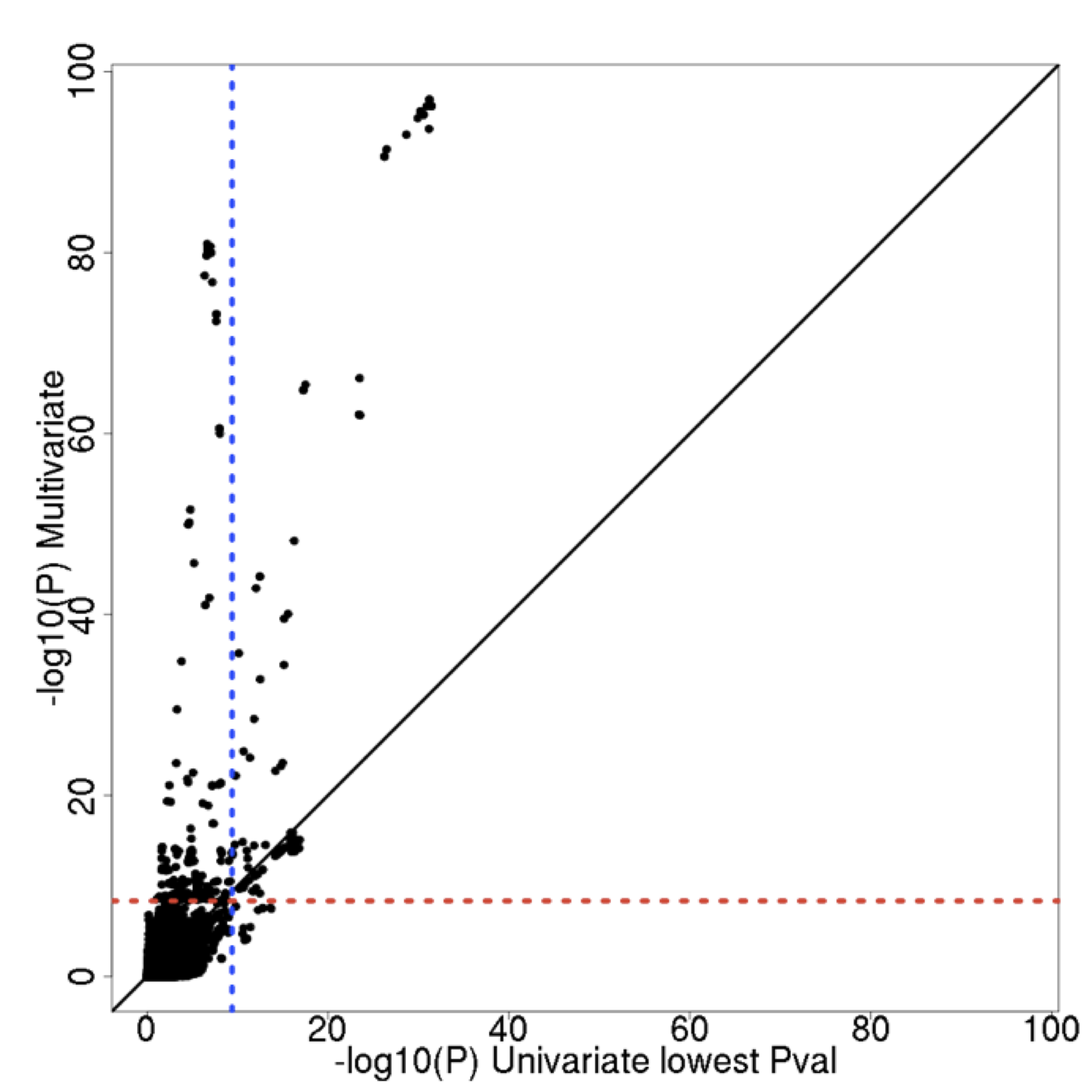

Supplement: Figure S2 — Comparison of P values from association testing of metabolic networks versus single metabolites. Testing multiple metabolites simultaneously shows an enrichment of low multivariate P values. Multivariate and univariate P values were compared across all 11 metabolic networks for the 2,406,682 SNPs in the YFS cohort. The different multiple testing burden are shown by dotted lines: horizontal red for multivariate and vertical blue for univariate testing. The univariate P value for a SNP was determined via the minimum after testing all single metabolites in a network. (TIFF) [file pgen.1002907.s002.tiff]

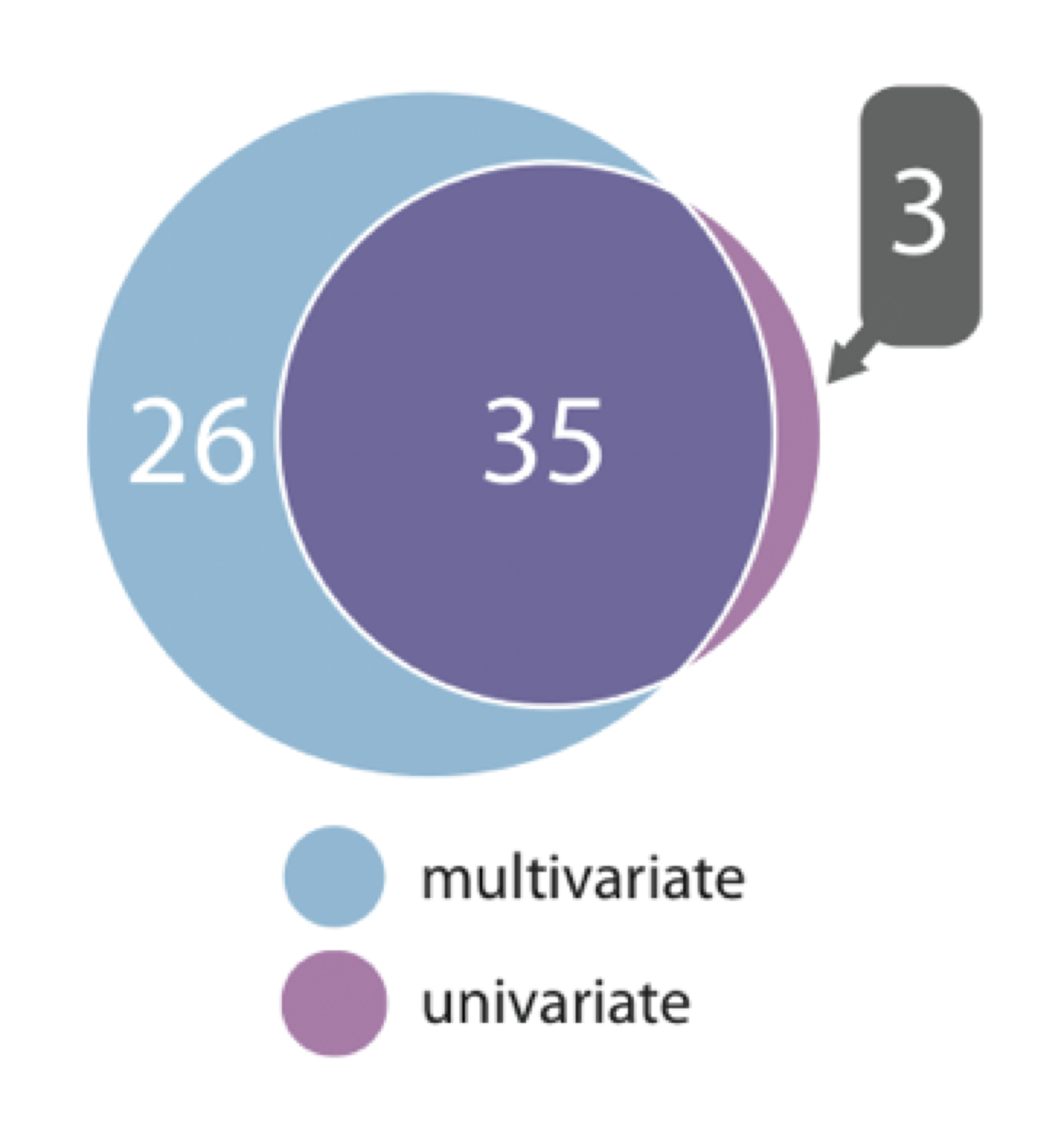

Supplement: Figure S3 — Associations detected between metabolic networks and loci previously associated with metabolism. (TIFF) [file pgen.1002907.s003.tiff]

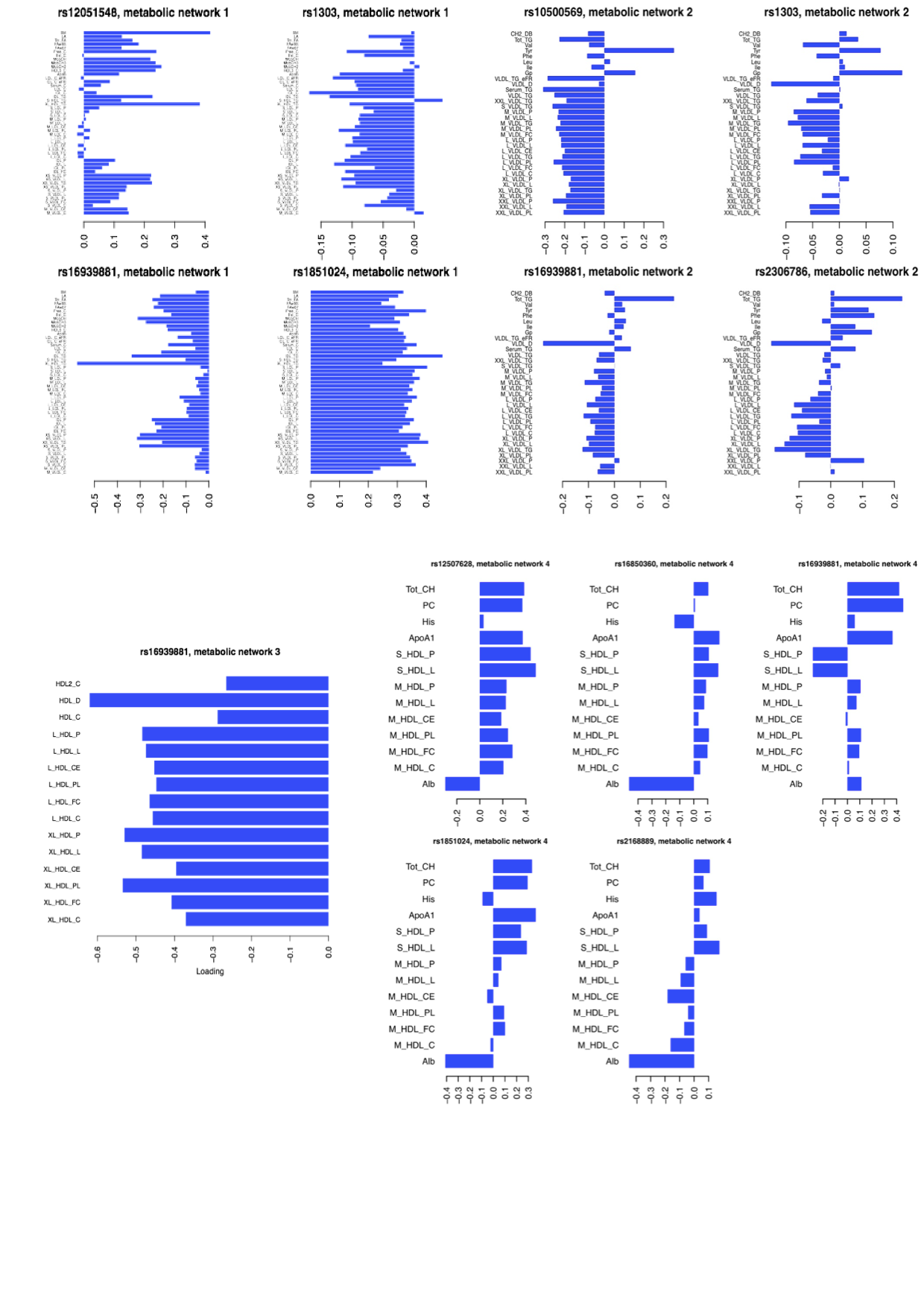

Supplement: Figure S4 — Loadings from multivariate metabolic network tests. (TIFF) [file pgen.1002907.s004.tiff]

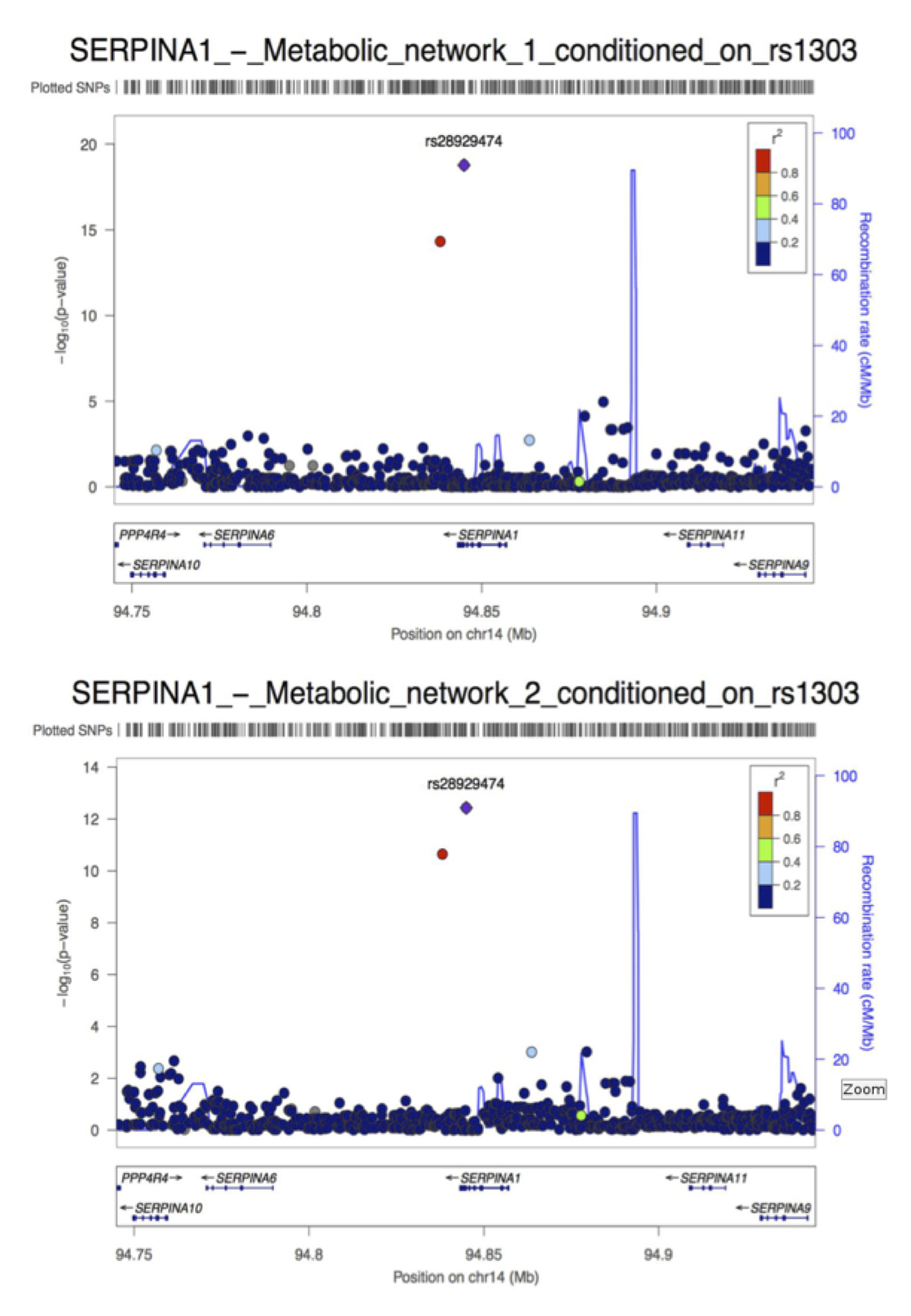

Supplement: Figure S5 — Conditional analysis of the SERPINA1 loci. (TIFF) [file pgen.1002907.s005.tifff]

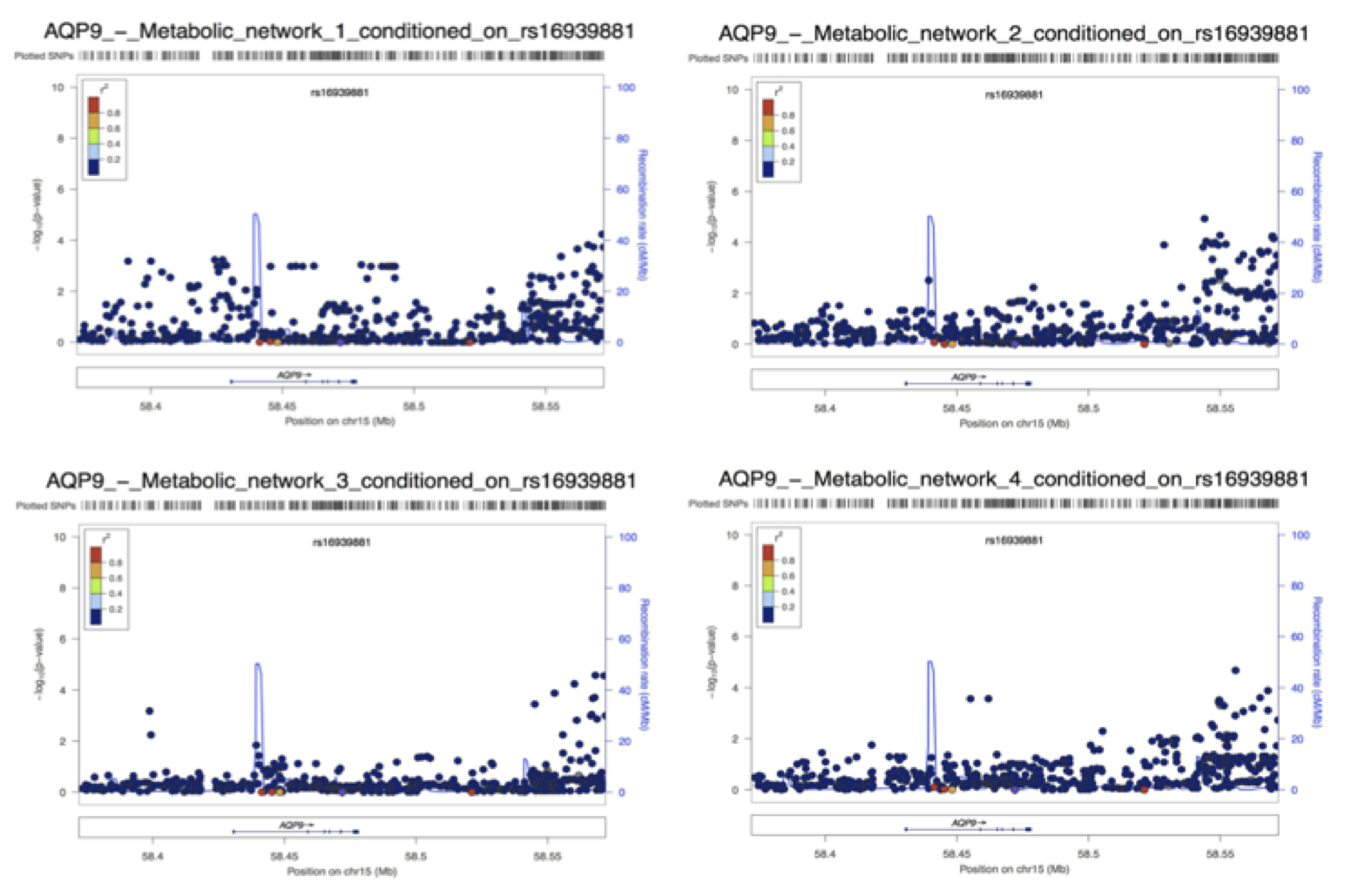

Supplement: Figure S6 — Conditional analysis of the AQP9 loci. (TIFF) [file pgen.1002907.s006.tiff]

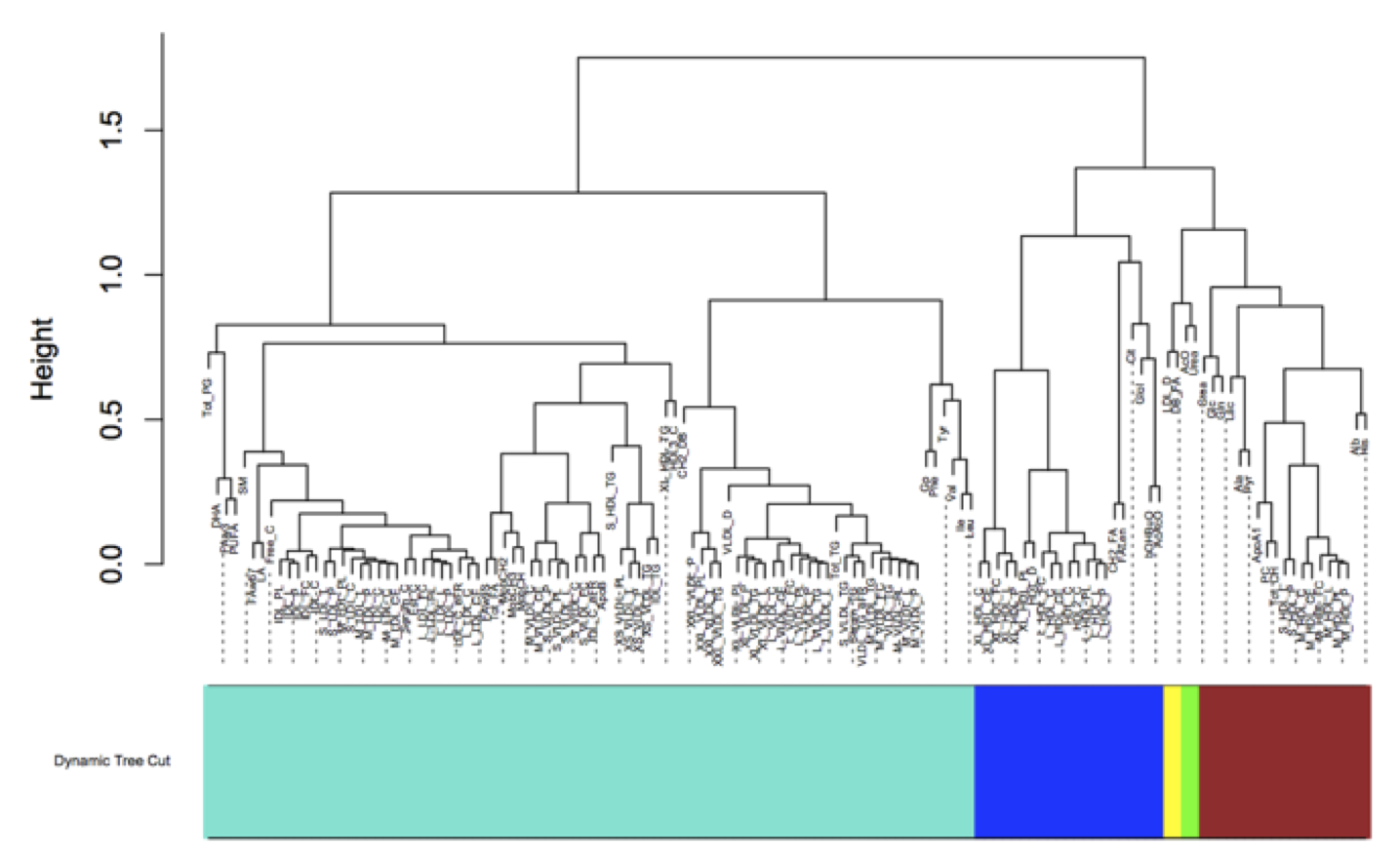

Supplement: Figure S7 — Hierarchical clustering and detection of 5 metabolite networks. (TIFF) [file pgen.1002907.s007.tiff]
