# Supplementary material for: Novel Loci for Metabolic Networks and Multi-Tissue Expression Studies Reveal Genes for Atherosclerosis
Source: PLoS Genet. 2012 Aug 16;8(8):e1002907. doi: 10.1371/journal.pgen.1002907 (PMC3420921; doi:10.1371/journal.pgen.1002907)

SECTION DESCRIBING METABOLIC NETWORKS

Metabolite Network 1

Composition of network (N = 49):

Metabolite abbreviation	Full description
M-VLDL-C	Total cholesterol in medium VLDL
M-VLDL-CE	Cholesterol esters in medium VLDL
S-VLDL-P	Concentration of small VLDL particles
S-VLDL-L	Total lipids in small VLDL
S-VLDL-PL	Phospholipids in small VLDL
S-VLDL-C	Total cholesterol in small VLDL
S-VLDL-FC	Free cholesterol in small VLDL
XS-VLDL-P	Concentration of very small VLDL particles
XS-VLDL-L	Total lipids in very small VLDL
XS-VLDL-PL	Phospholipids in very small VLDL
XS-VLDL-TG	Triglycerides in very small VLDL
IDL-P	Concentration of IDL particles
IDL-L	Total lipids in IDL
IDL-PL	Phospholipids in IDL
IDL-C	Total cholesterol in IDL
IDL-FC	Free cholesterol in IDL
IDL-TG	Triglycerides in IDL
L-LDL-P	Concentration of large LDL particles
L-LDL-L	Total lipids in large LDL
L-LDL-PL	Phospholipids in large LDL
L-LDL-C	Total cholesterol in large LDL
L-LDL-CE	Cholesterol esters in large LDL
L-LDL-FC	Free cholesterol in large LDL
M-LDL-P	Concentration of medium LDL particles
M-LDL-L	Total lipids in medium LDL
M-LDL-PL	Phospholipids in medium LDL
M-LDL-C	Total cholesterol in medium LDL
M-LDL-CE	Cholesterol esters in medium LDL
S-LDL-P	Concentration of small LDL particles
S-LDL-L	Total lipids in small LDL
S-LDL-C	Total cholesterol in small LDL
XL-HDL-TG	Triglycerides in very large HDL
S-HDL-TG	Triglycerides in small HDL
LDL-C	Total cholesterol in LDL
Serum-C	Serum total cholesterol
MobCH	Double bond protons of mobile lipids
MobCH2	CH2 groups of mobile lipids
MobCH3	CH3 groups of mobile lipids
Est-C	Esterified cholesterol
Free-C	Free cholesterol
FAw67	Omega-6 and -7 fatty acids
FAw9S	Omega-9 and saturated fatty acids
Tot-FA	Total fatty acids
LA	18:2, linoleic acid (LA)

SM	Sphingomyelins and other sphingolipids
IDL-C-eFR	Total cholesterol in IDL (Lipido)
LDL-C-eFR	Total cholesterol in LDL (Lipido)
ApoB	Apolipoprotein B (Lipido)
HDL3-C	Total cholesterol in HDL3 (Lipido)

Intra-correlation of network:

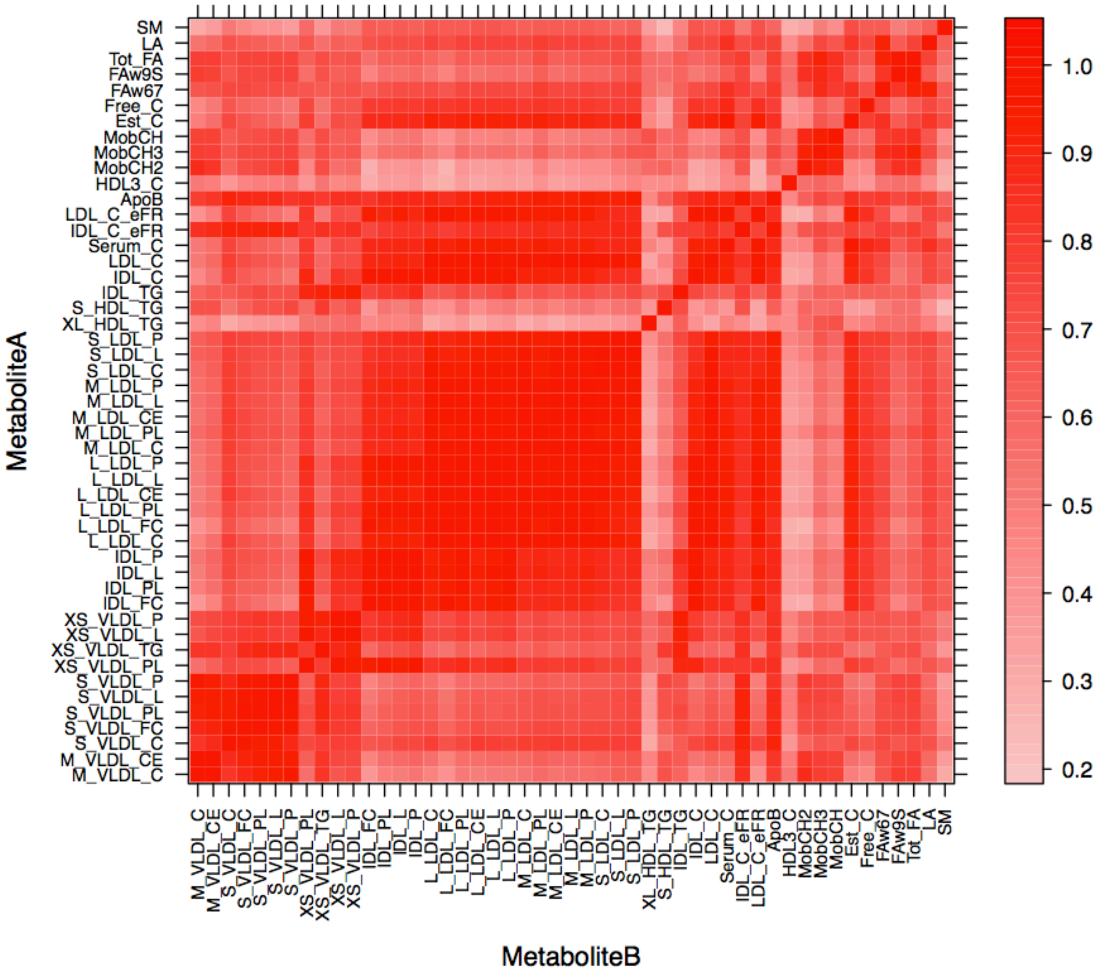

QQ plots:

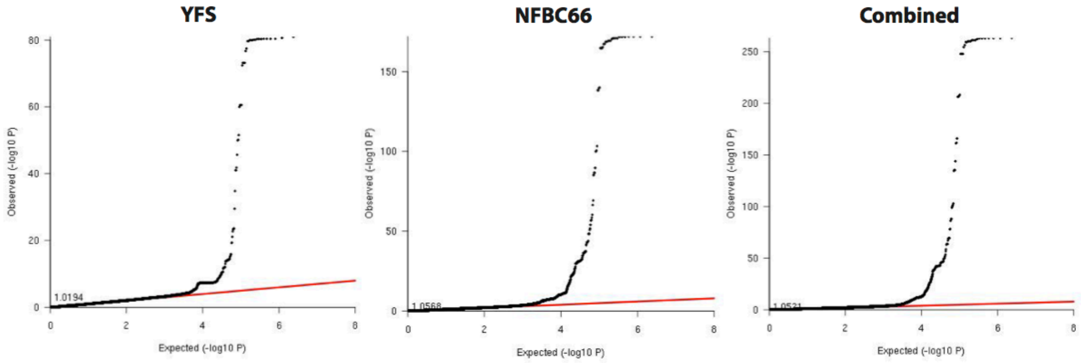

Comparison of multivariate and univariate *P* values:

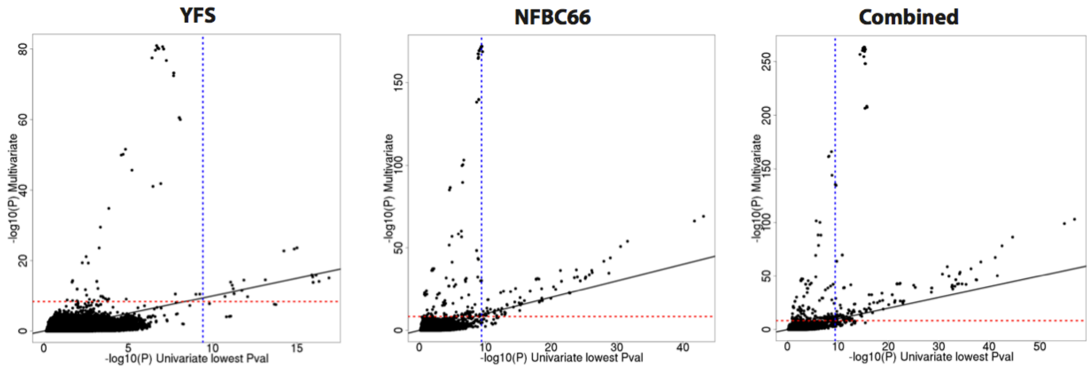

Genome-wide Manhattan plot for combined analysis:

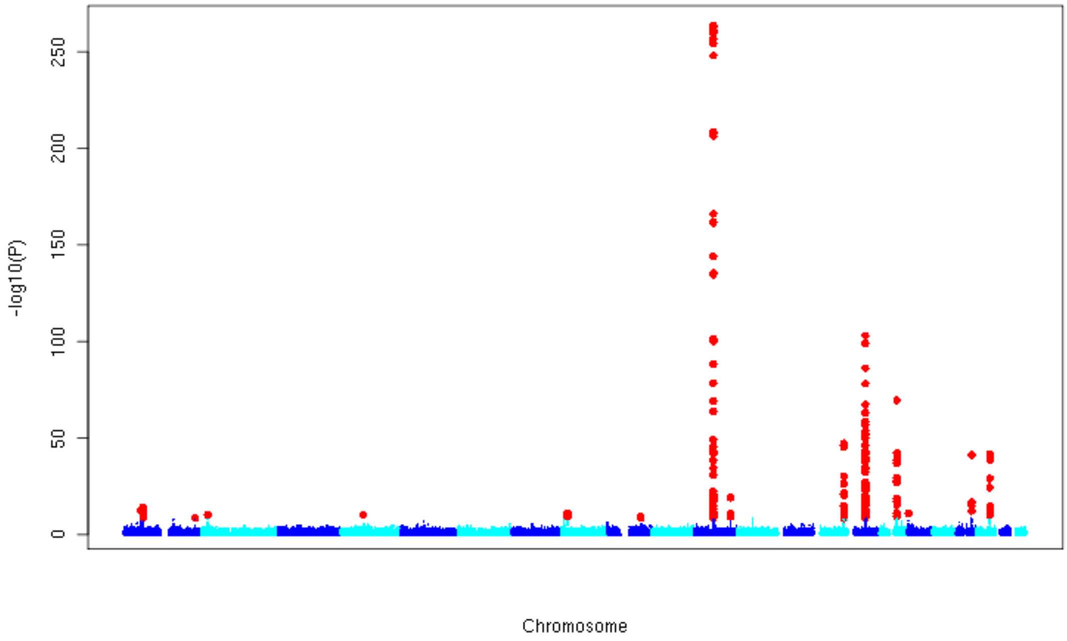

Regional Manhattan plots for combined analysis of novel regions:

AQP9 locus – Metabolite network 1

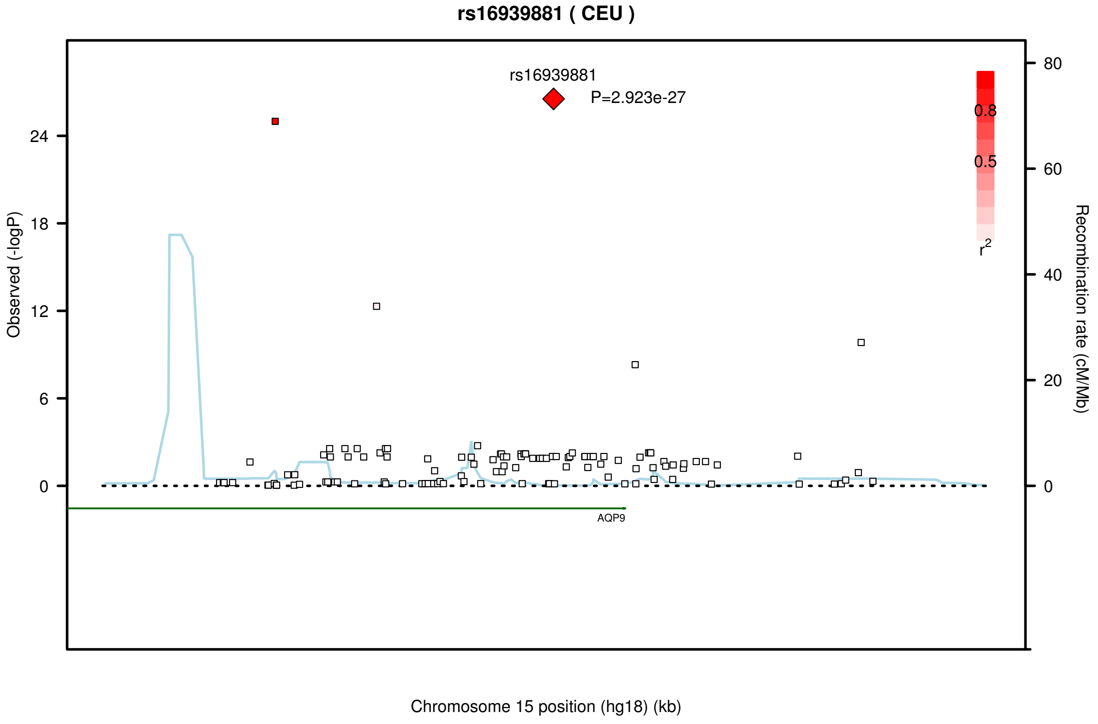

GC locus – Metabolite network 1

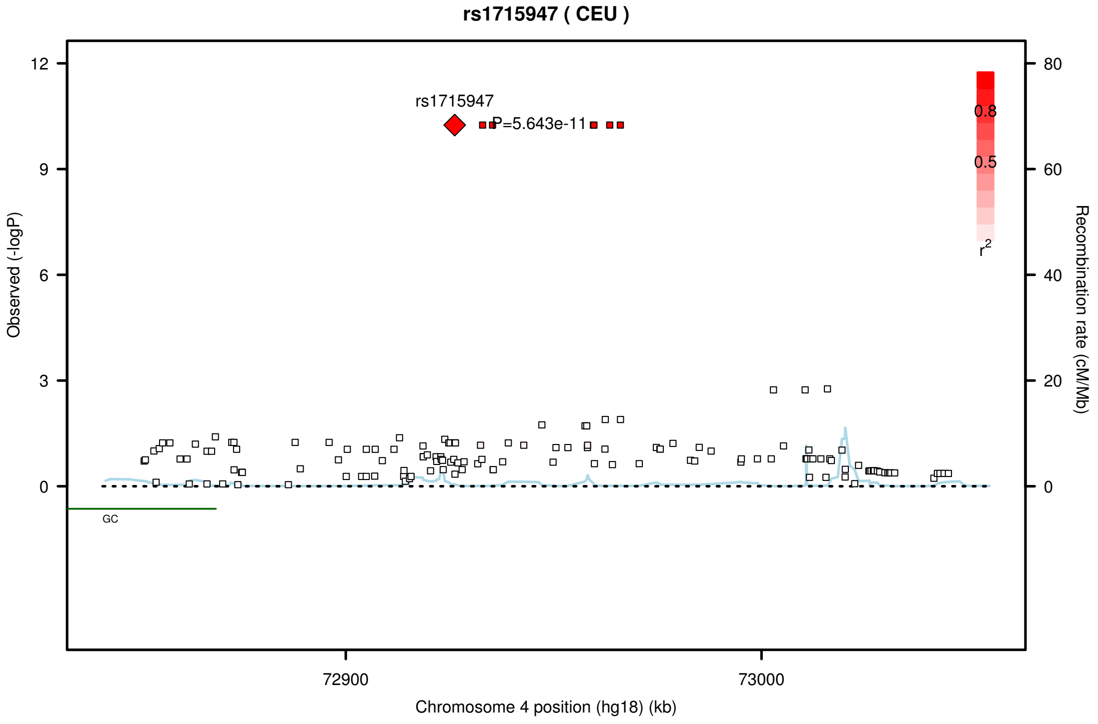

SERPINA1 **locus – Metabolite network 1**

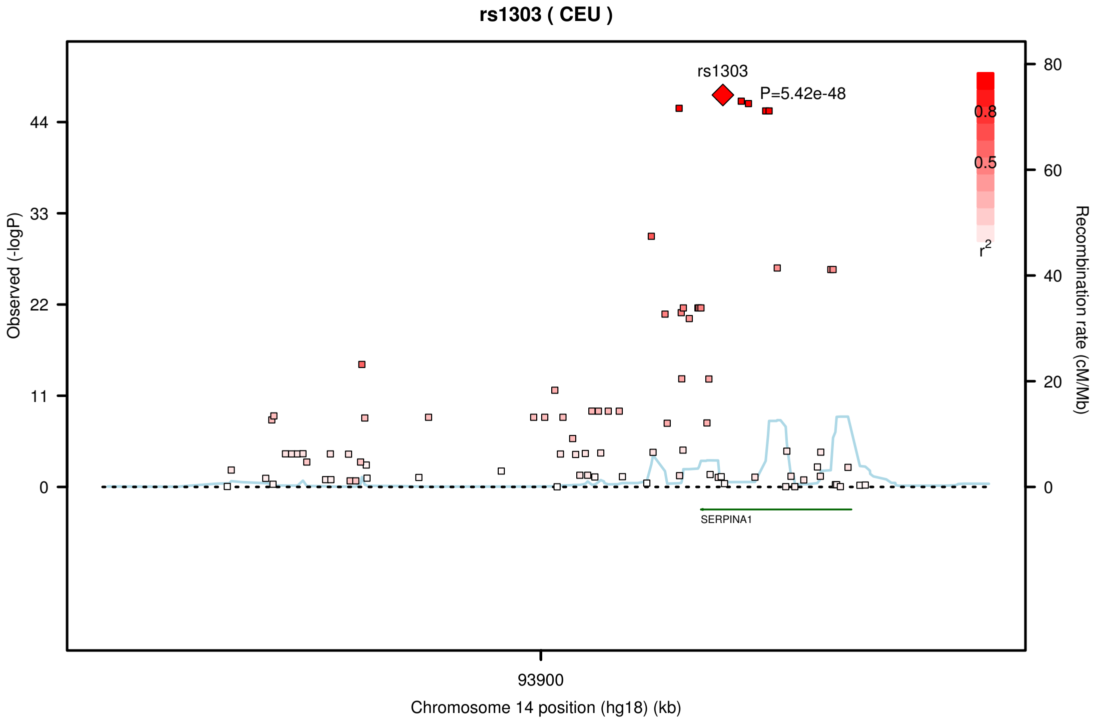

Metabolite Network 2

Composition of network (N = 33):

Metabolite abbreviation	Full description
XXL-VLDL-P	Concentration of chylomicrons and extremely large VLDL particles
XXL-VLDL-L	Total lipids in chylomicrons and extremely large VLDL
XXL-VLDL-PL	Phospholipids in chylomicrons and extremely large VLDL
XXL-VLDL-TG	Triglycerides in chylomicrons and extremely large VLDL
XL-VLDL-P	Concentration of very large VLDL particles
XL-VLDL-L	Total lipids in very large VLDL
XL-VLDL-PL	Phospholipids in very large VLDL
XL-VLDL-TG	Triglycerides in very large VLDL
L-VLDL-P	Concentration of large VLDL particles
L-VLDL-L	Total lipids in large VLDL
L-VLDL-PL	Phospholipids in large VLDL
L-VLDL-C	Total cholesterol in large VLDL
L-VLDL-CE	Cholesterol esters in large VLDL
L-VLDL-FC	Free cholesterol in large VLDL
L-VLDL-TG	Triglycerides in large VLDL
M-VLDL-P	Concentration of medium VLDL particles
M-VLDL-L	Total lipids in medium VLDL
M-VLDL-PL	Phospholipids in medium VLDL
M-VLDL-FC	Free cholesterol in medium VLDL
M-VLDL-TG	Triglycerides in medium VLDL
S-VLDL-TG	Triglycerides in small VLDL
VLDL-TG	Triglycerides in VLDL
Serum-TG	Serum total triglycerides
Gp	Glycoprotein acetyls, mainly a1-acid glycoprotein
Ile	Isoleucine
Leu	Leucine
Phe	Phenylalanine
Tyr	Tyrosine
Val	Valine
Tot-TG	Total triglycerides
VLDL-D	Mean diameter for VLDL particles
VLDL-TG-eFR	Triglycerides in VLDL (Lipido)
CH2/DB	Average number of methylene groups per a double bond

Intra-correlation of network:

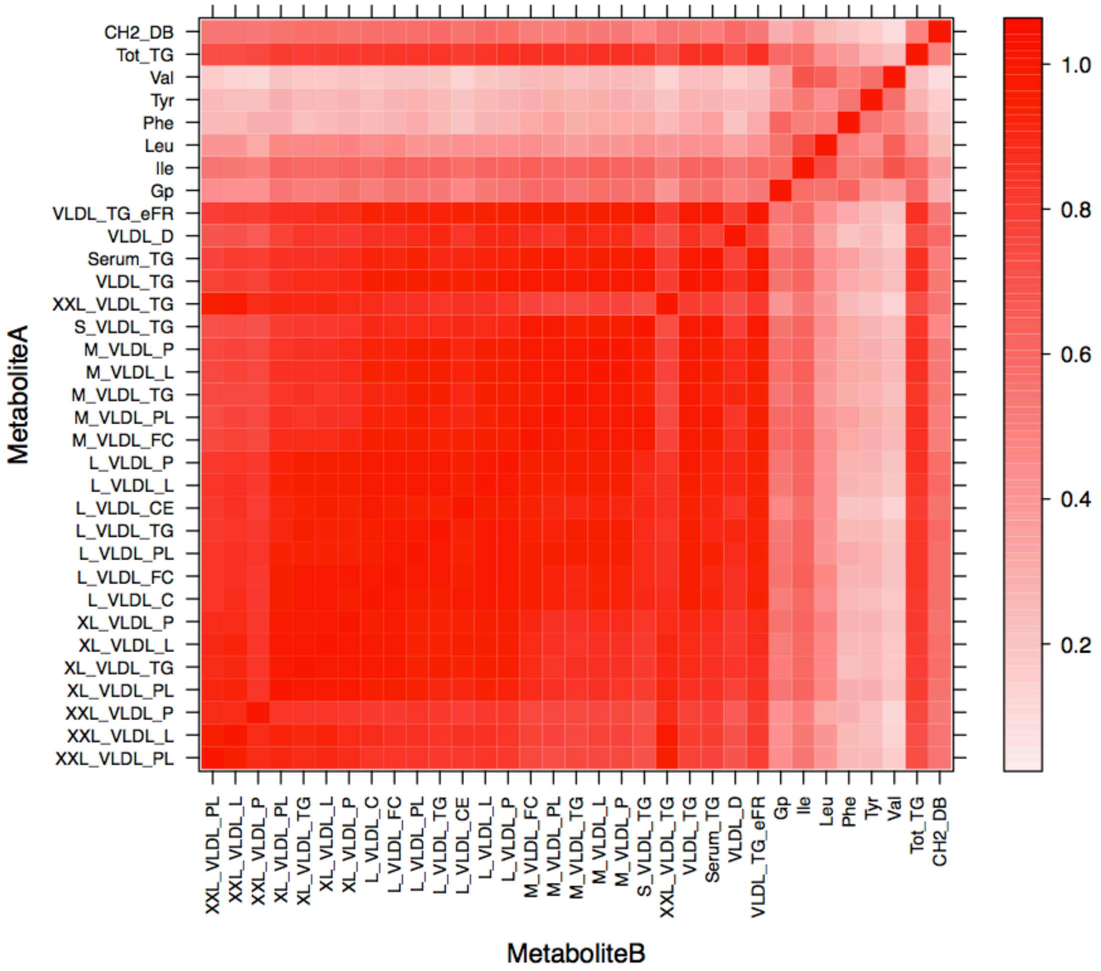

QQ plots:

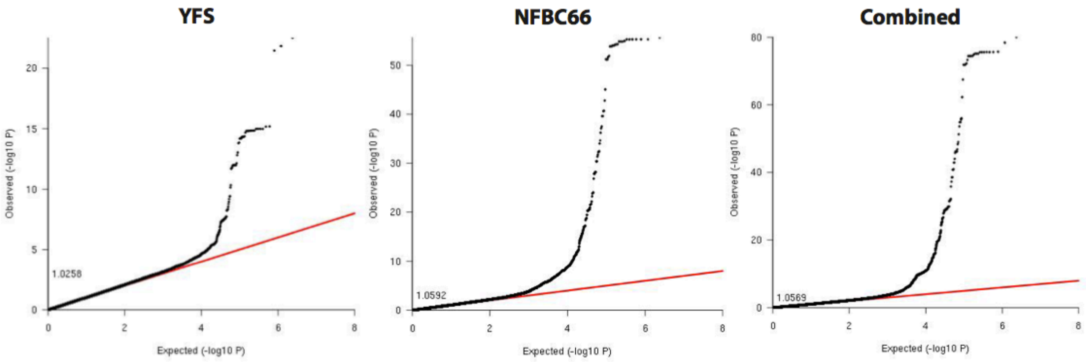

Comparison of multivariate and univariate *P* values:

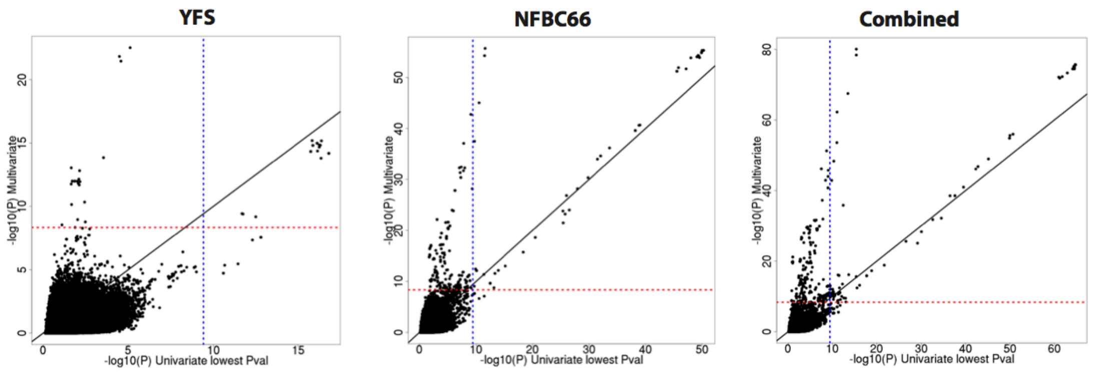

Genome-wide Manhattan plot for combined analysis:

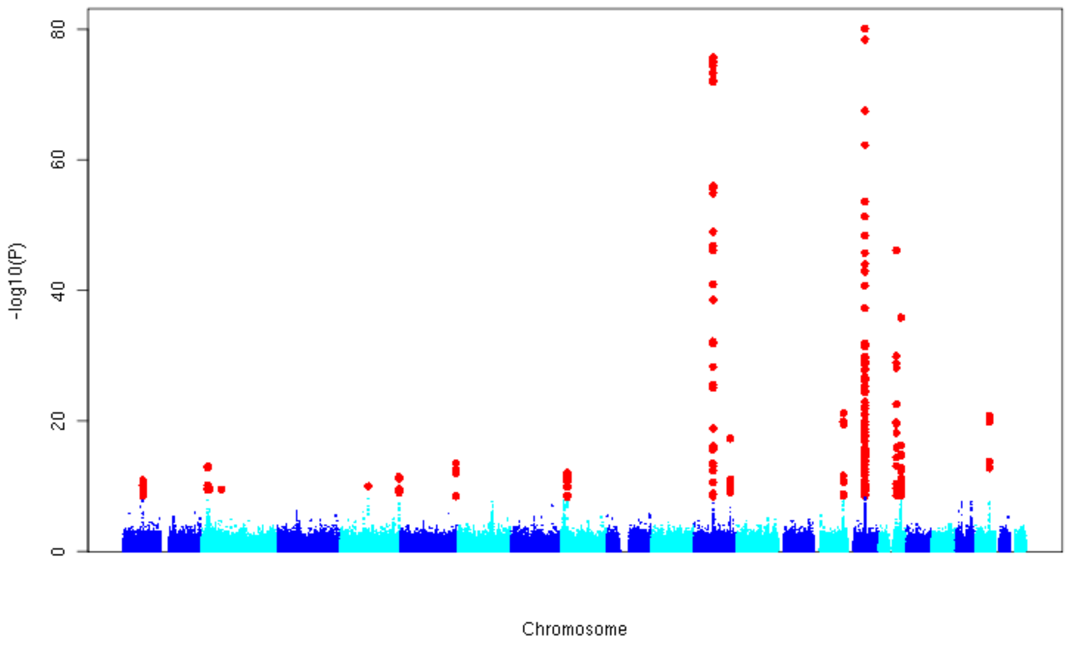

Regional Manhattan plots for combined analysis of novel regions:

AQP9 locus – Metabolite network 2

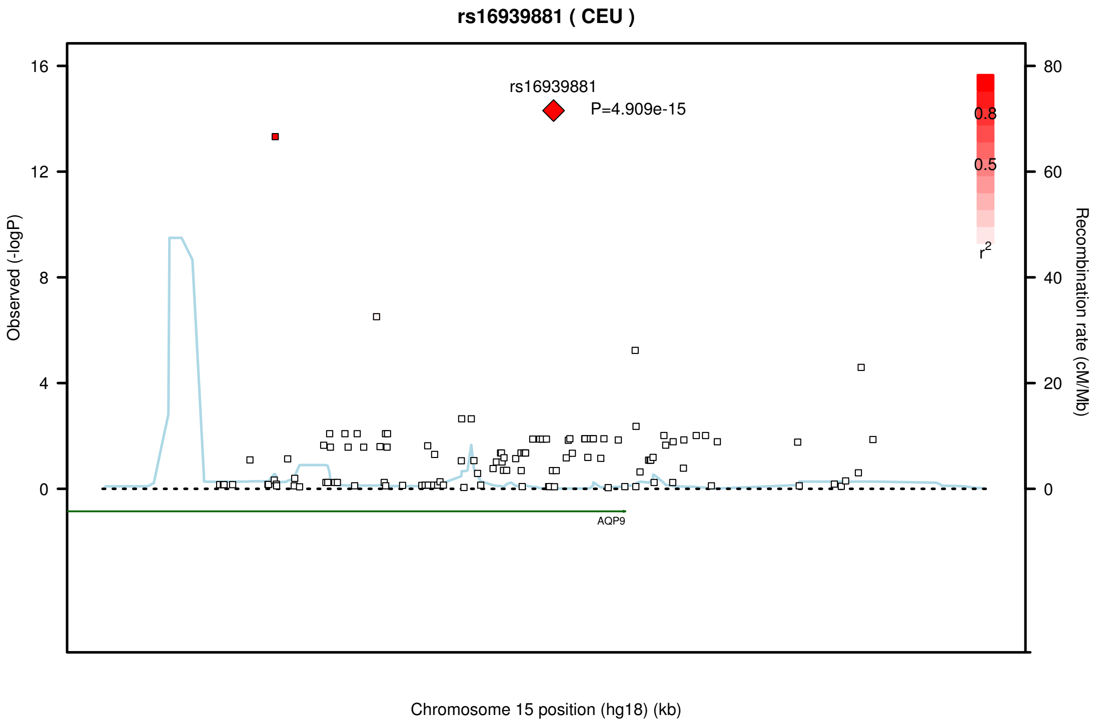

MYO1E, CCNB2, RNF111 locus – Metabolite network 2

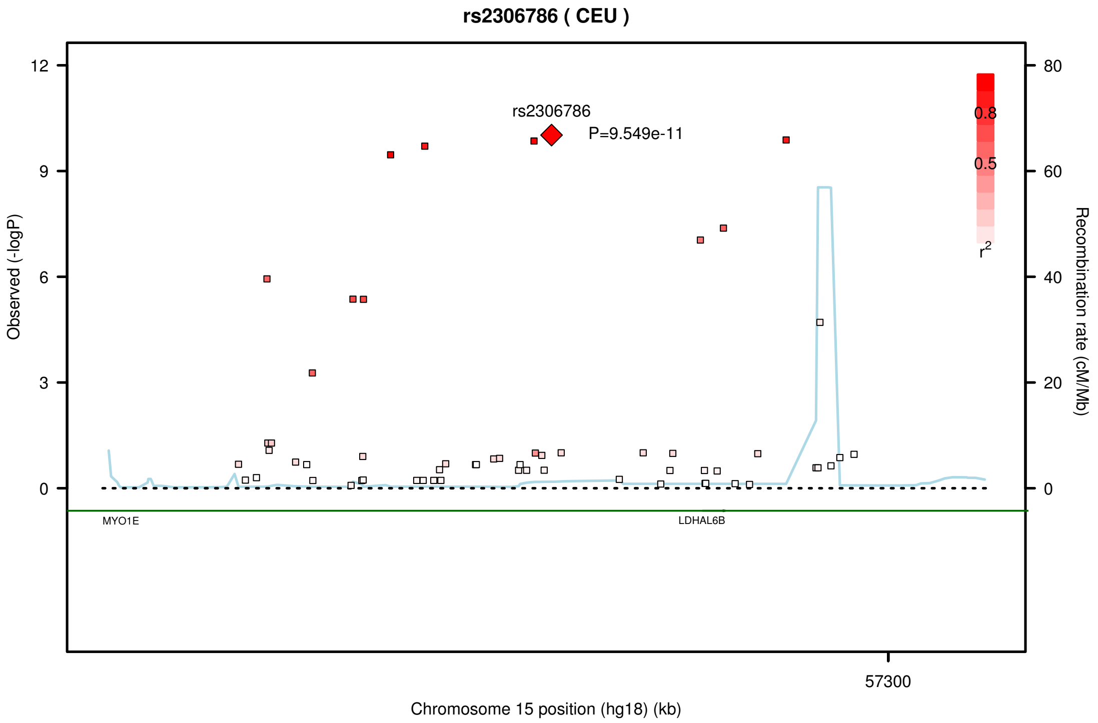

SERPINA1 **locus – Metabolite network 2**

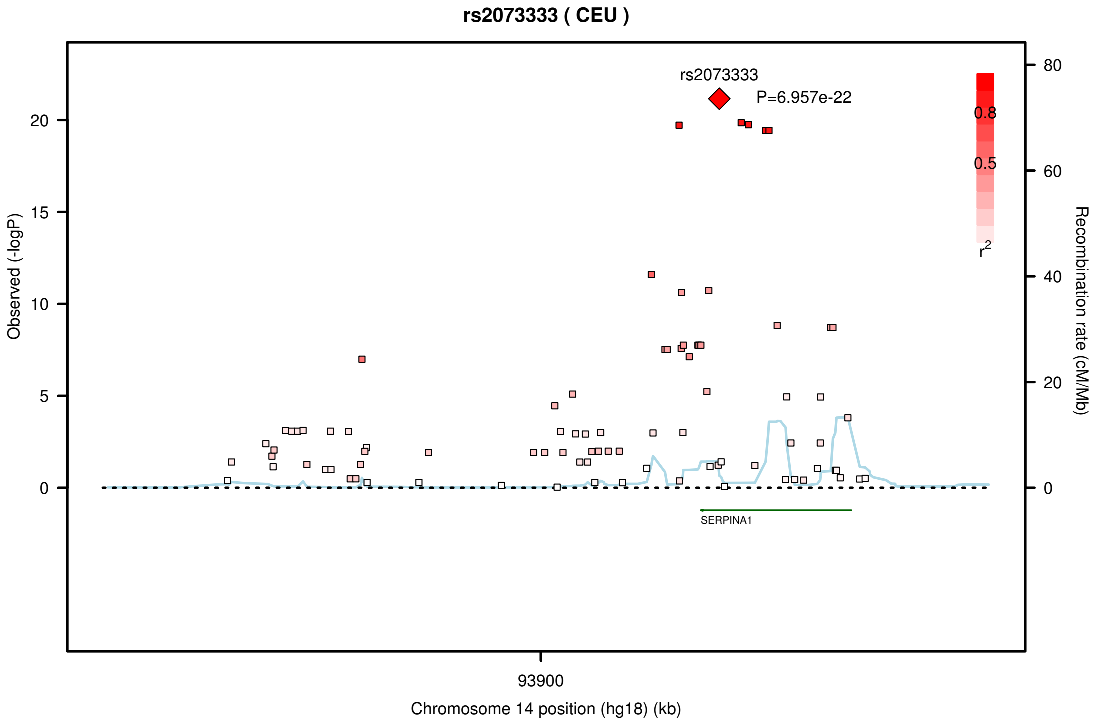

ZFHX3 **locus – Metabolite network 2**

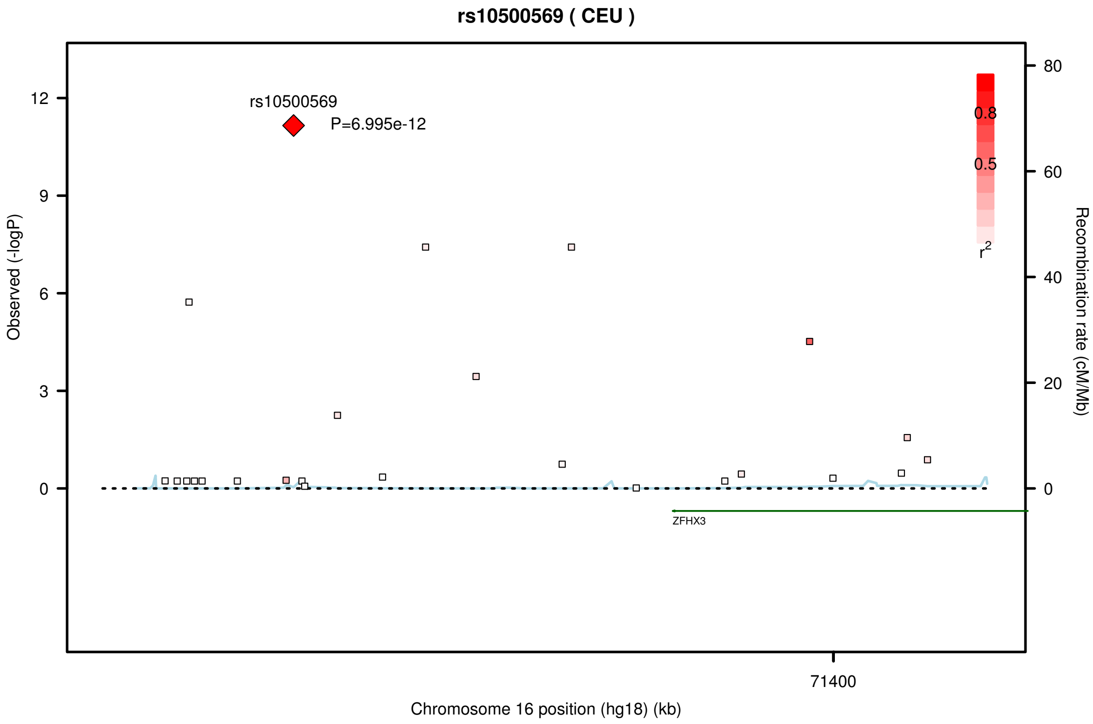

Metabolite Network 3

Composition of network (N = 15):

**Metabolite abbreviation**	**Full description**
XL-HDL-P	Concentration of very large HDL particles
XL-HDL-L	Total lipids in very large HDL
XL-HDL-PL	Phospholipids in very large HDL
XL-HDL-C	Total cholesterol in very large HDL
XL-HDL-CE	Cholesterol esters in very large HDL
XL-HDL-FC	Free cholesterol in very large HDL
L-HDL-P	Concentration of large HDL particles
L-HDL-L	Total lipids in large HDL
L-HDL-PL	Phospholipids in large HDL
L-HDL-C	Total cholesterol in large HDL
L-HDL-CE	Cholesterol esters in large HDL
L-HDL-FC	Free cholesterol in large HDL
HDL-C	Total cholesterol in HDL
HDL-D	Mean diameter for HDL particles
HDL2-C	Total cholesterol in HDL2 (Lipido)

Intra-correlation of network:

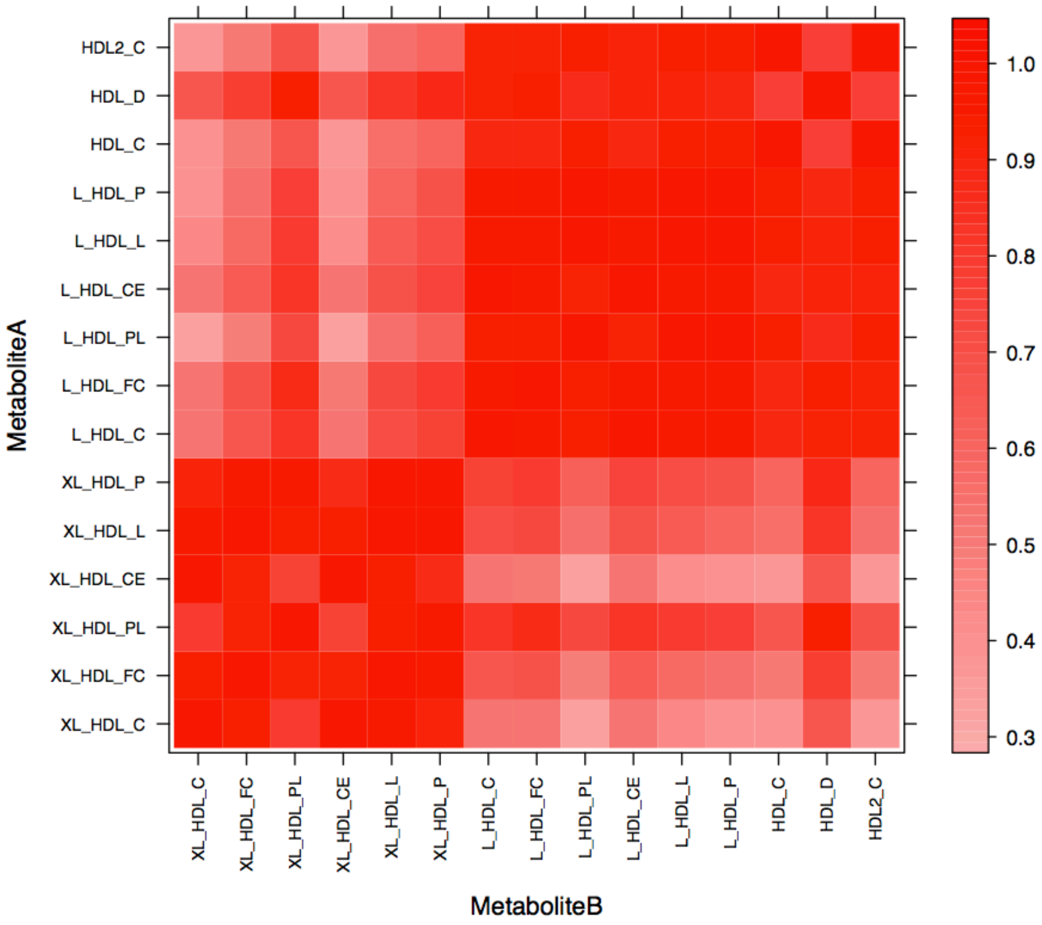

QQ plots:

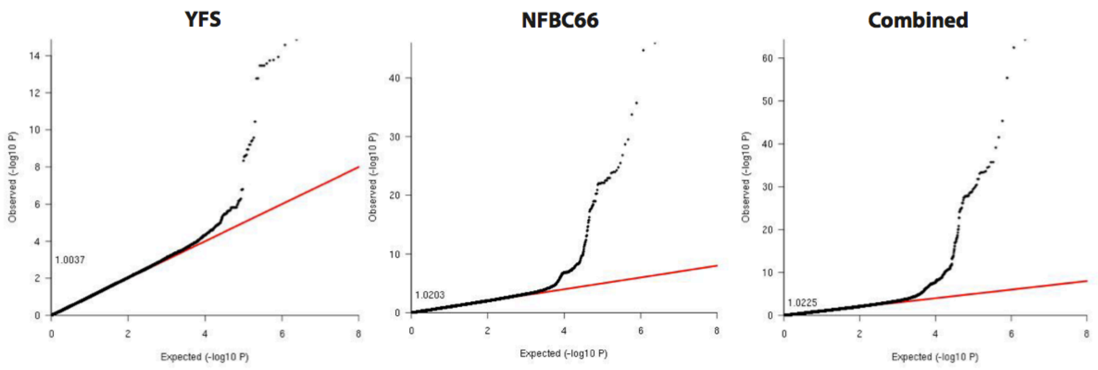

Comparison of multivariate and univariate *P* values from combined analysis:

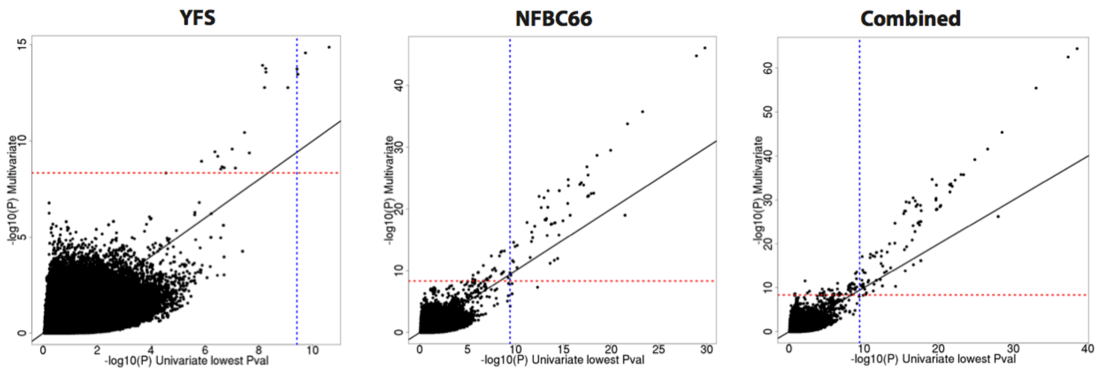

Genome-wide Manhattan plot for combined analysis:

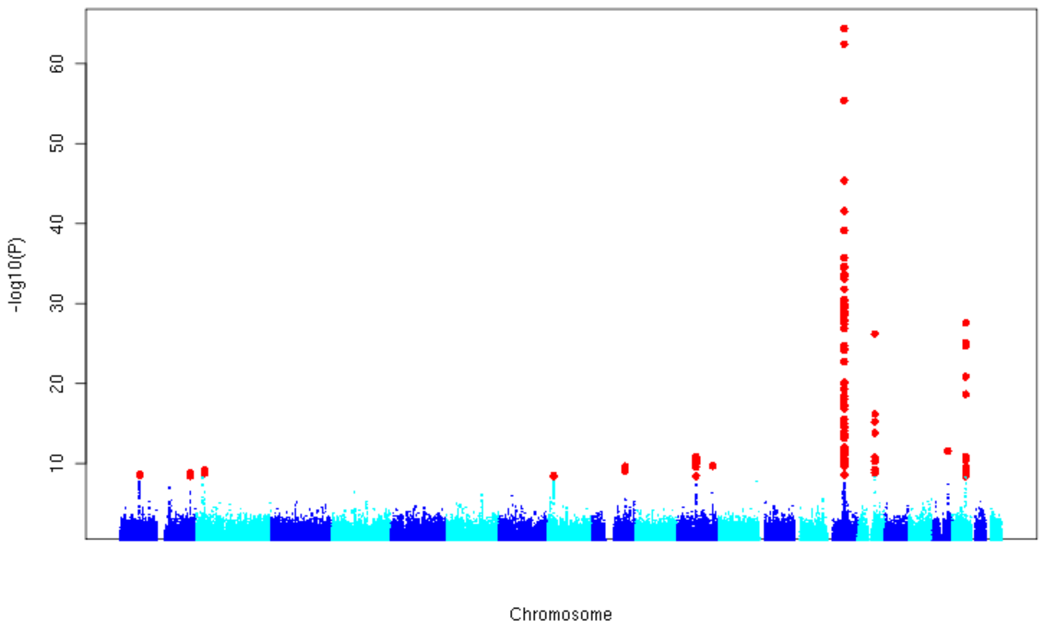

Regional Manhattan plots for combined analysis of novel regions:

AQP9 locus – Metabolite network 3

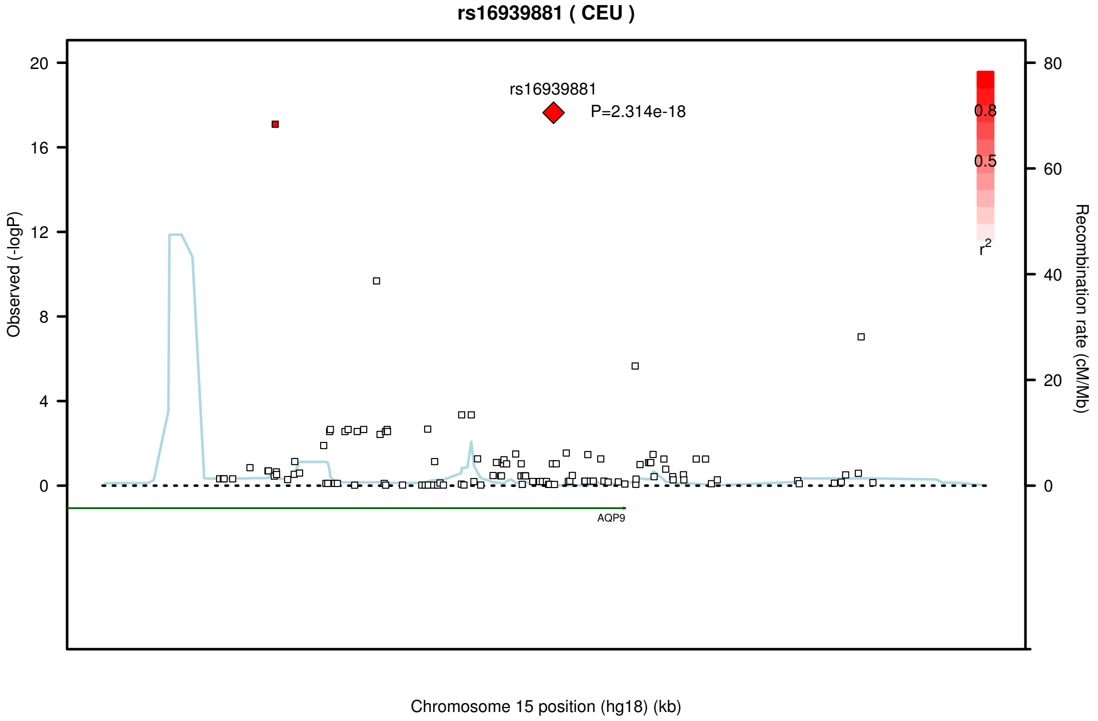

Metabolite Network 4

Composition of network (N = 13):

Metabolite abbreviation	Full description
M-HDL-P	Concentration of medium HDL particles
M-HDL-L	Total lipids in medium HDL
M-HDL-PL	Phospholipids in medium HDL
M-HDL-C	Total cholesterol in medium HDL
M-HDL-CE	Cholesterol esters in medium HDL
M-HDL-FC	Free cholesterol in medium HDL
S-HDL-P	Concentration of small HDL particles
S-HDL-L	Total lipids in small HDL
Alb	Albumin
His	Histidine
PC	Phosphatidylcholine and other cholines
Tot-CH	Total cholines (and other N-trimethyl compounds)
ApoA1	Apolipoprotein A-I (Lipido)

Intra-correlation of network:

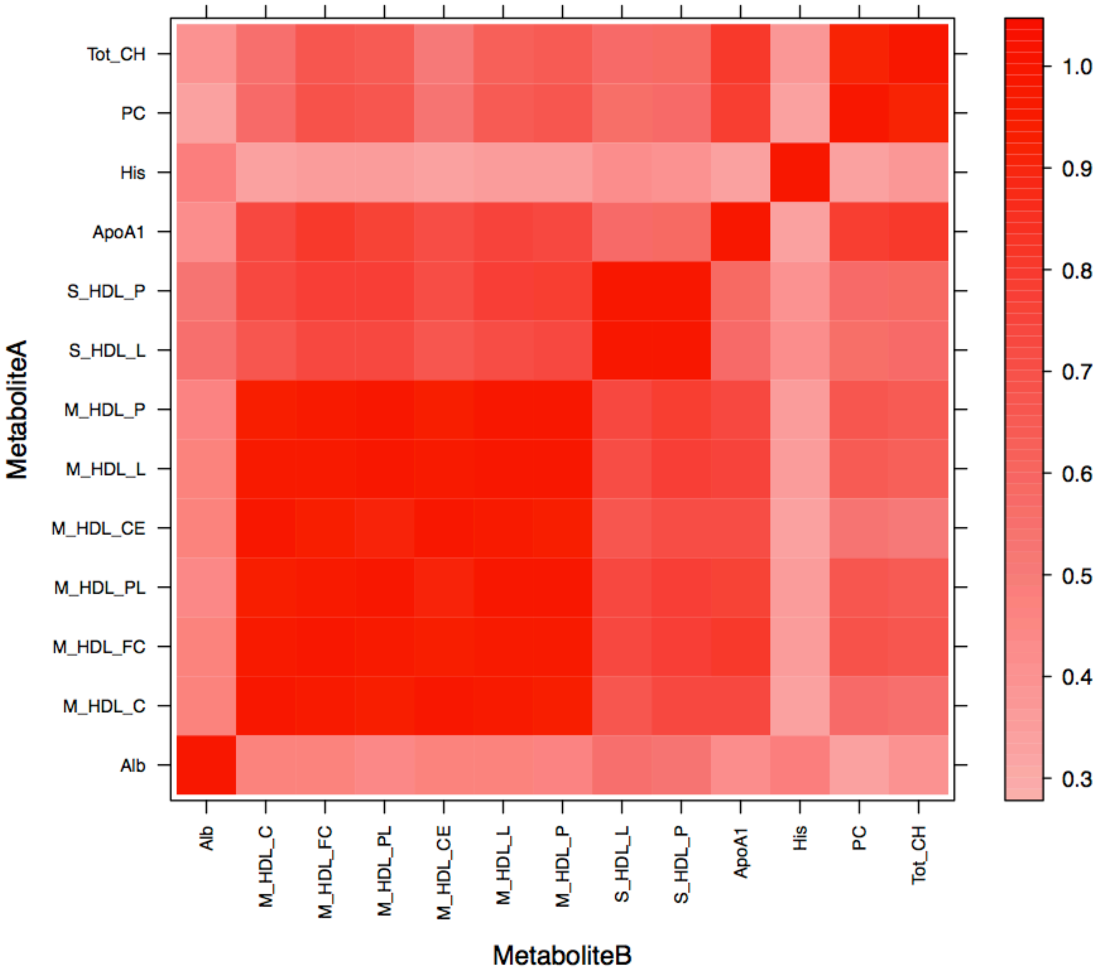

QQ plots:

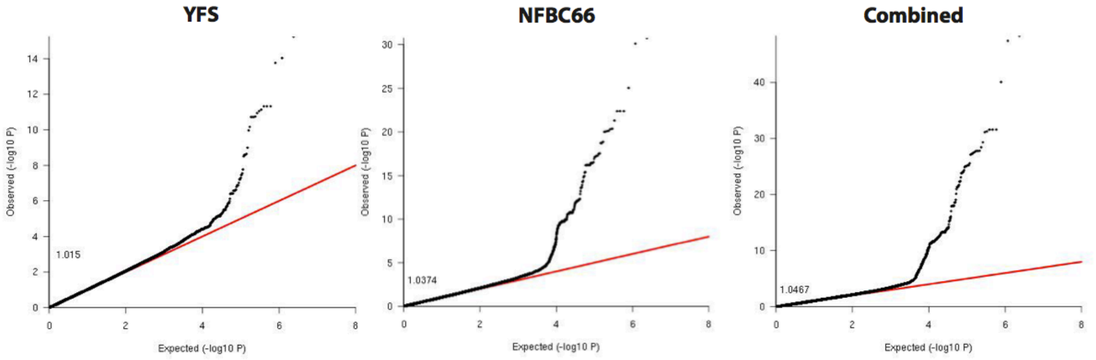

Comparison of multivariate and univariate *P* values:

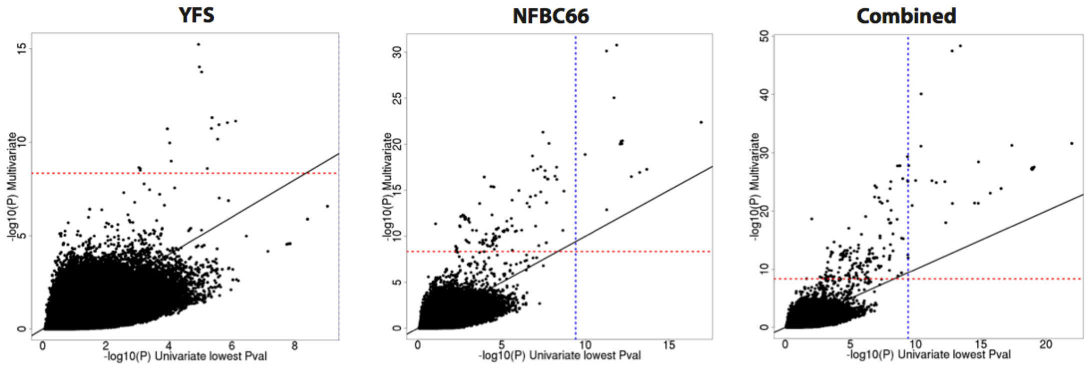

Genome-wide Manhattan plot for combined analysis:

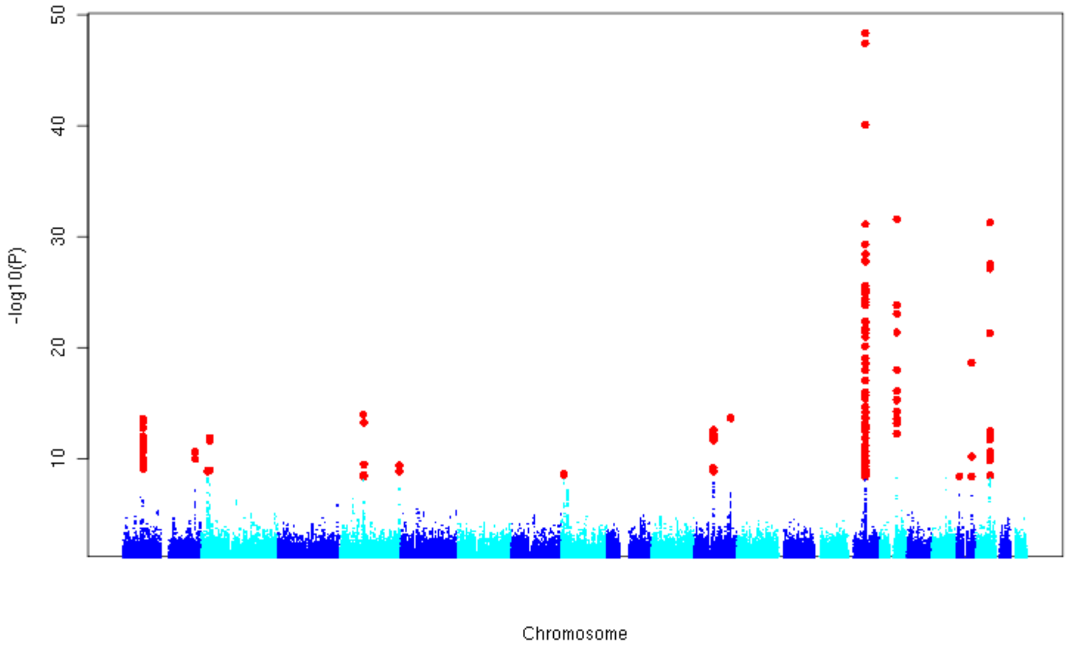

Regional Manhattan plots for combined analysis of novel regions:

***AQP9* locus – Metabolite network 4**

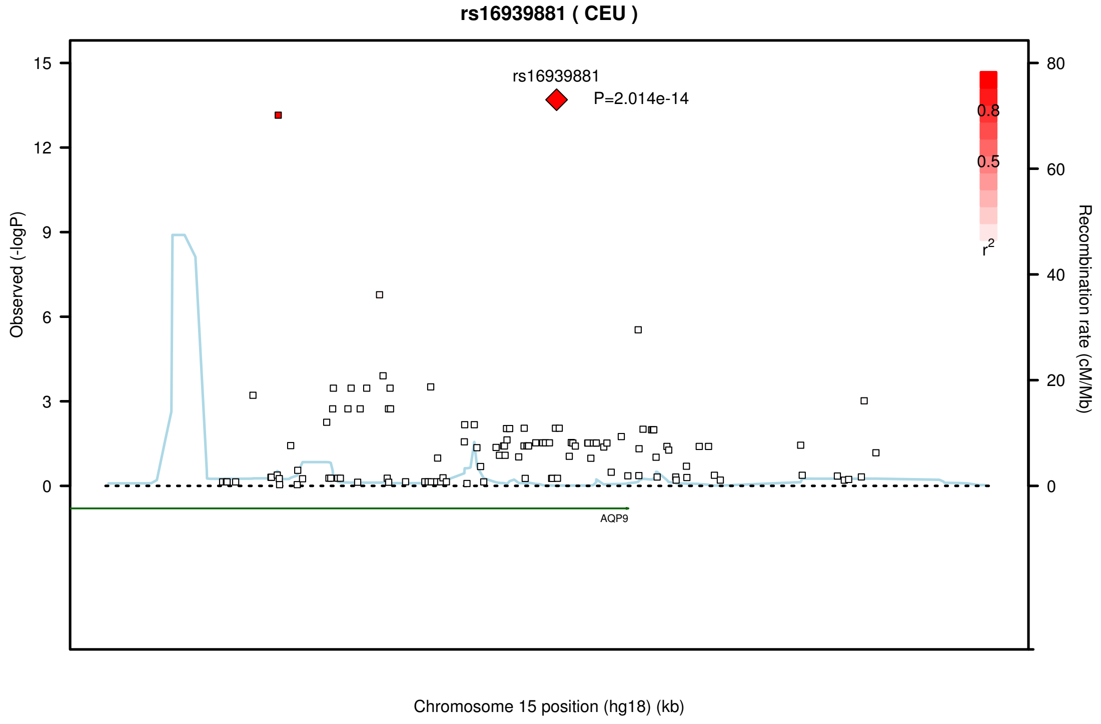

***EREG* locus – Metabolite network 4**

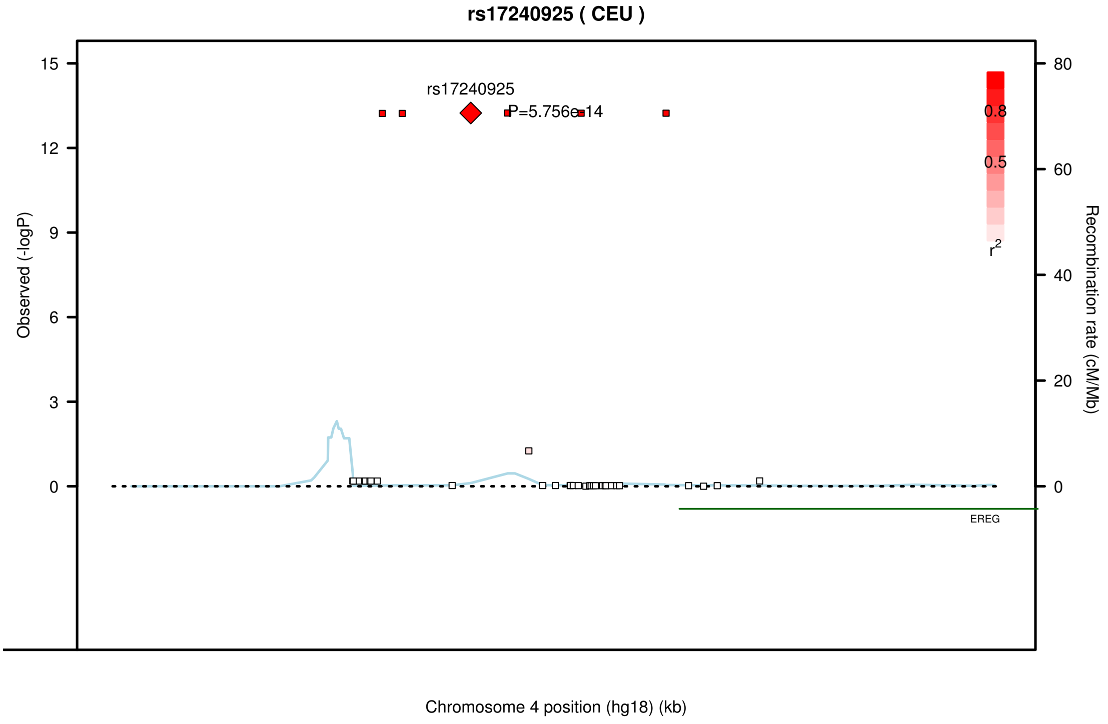

GC locus – Metabolite network 4

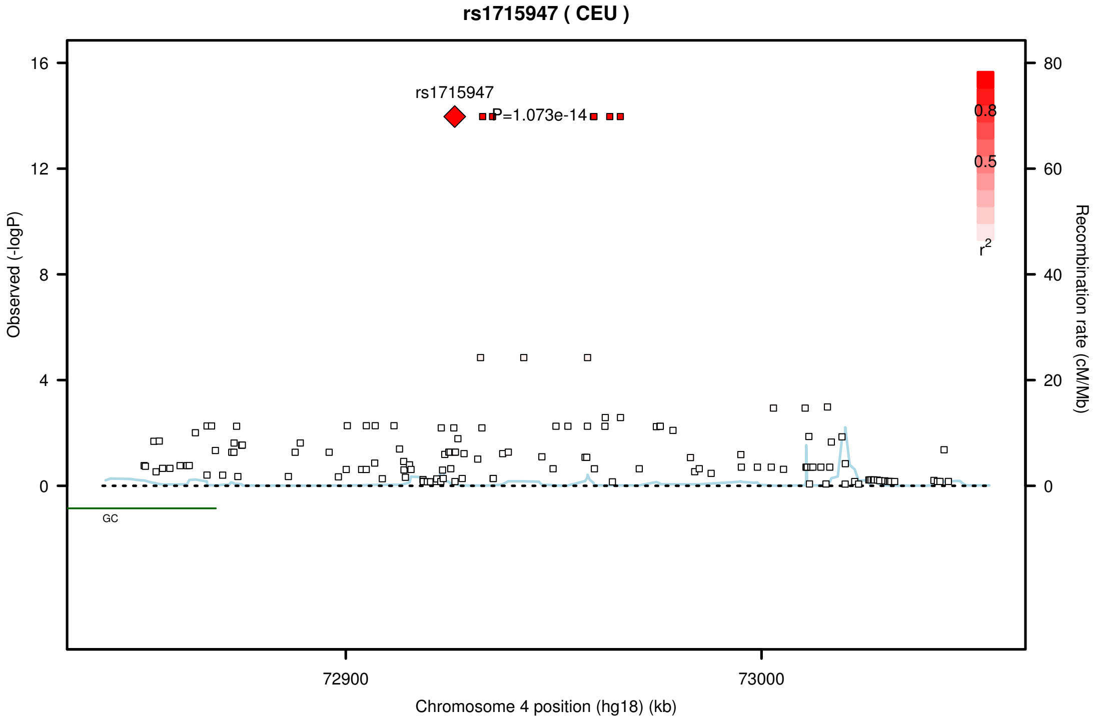

PF4, PPBP, CXCL5 locus – Metabolite network 4

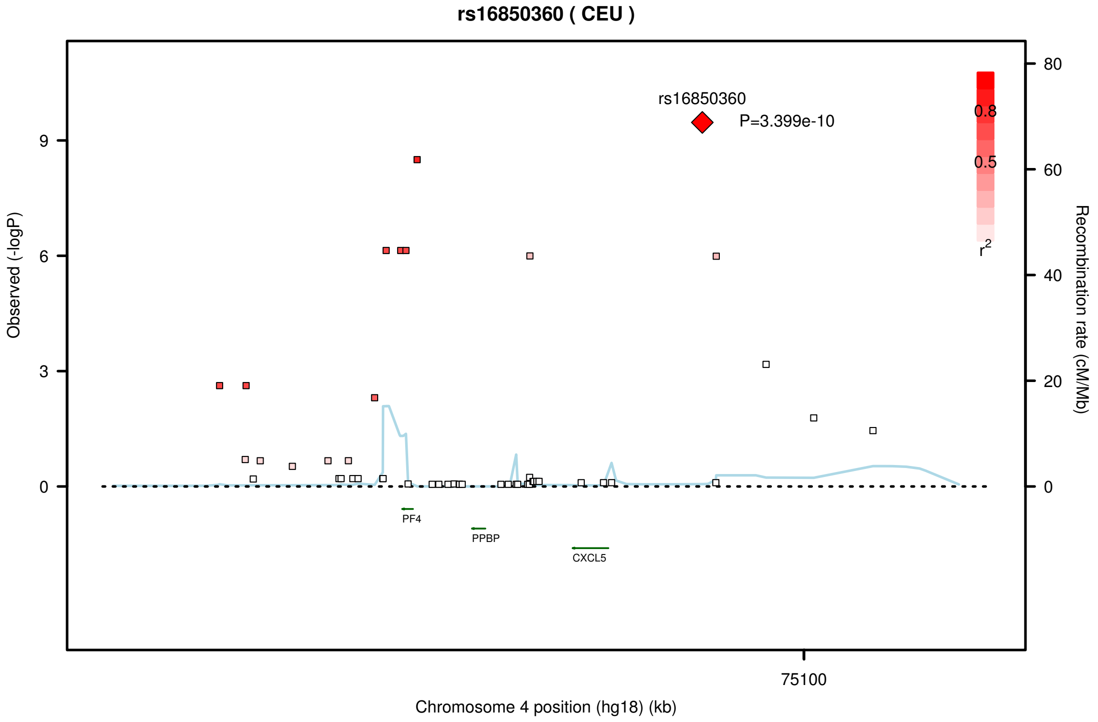

Metabolite Network 5

Composition of network (N = 4):

Metabolite abbreviation	Full description
FAw3	Omega-3 fatty acids
PUFA	Other polyunsaturated fatty acids than 18:2
DHA	22:6, docosahexaenoic acid (DHA)
Tot-PG	Total phosphoglycerides

Intra-correlation of network:

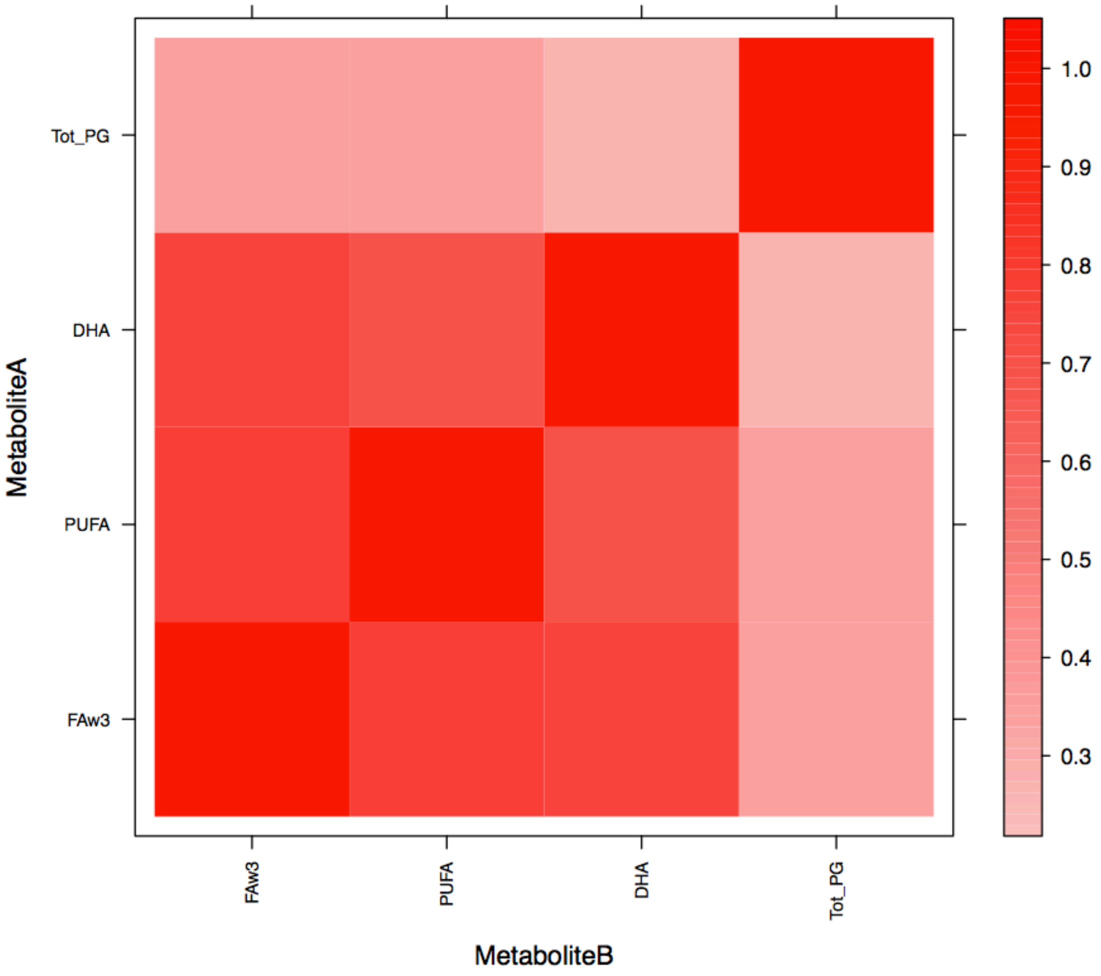

QQ plots:

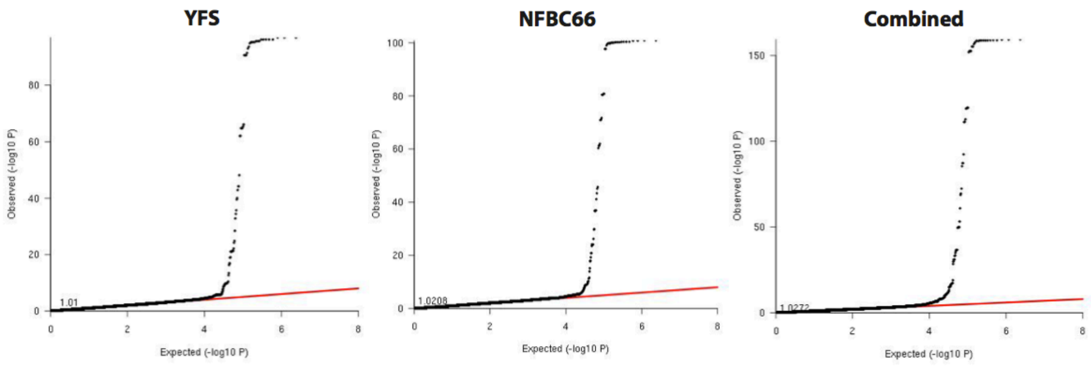

Comparison of multivariate and univariate *P* values:

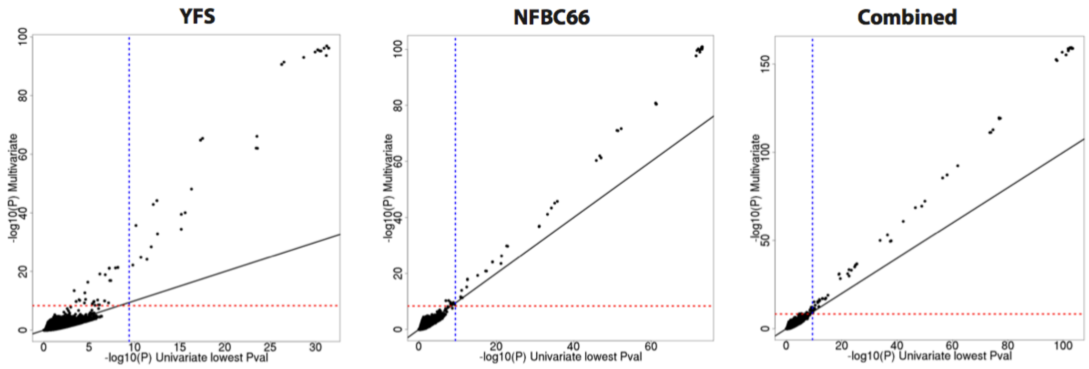

Genome-wide Manhattan plot for combined analysis:

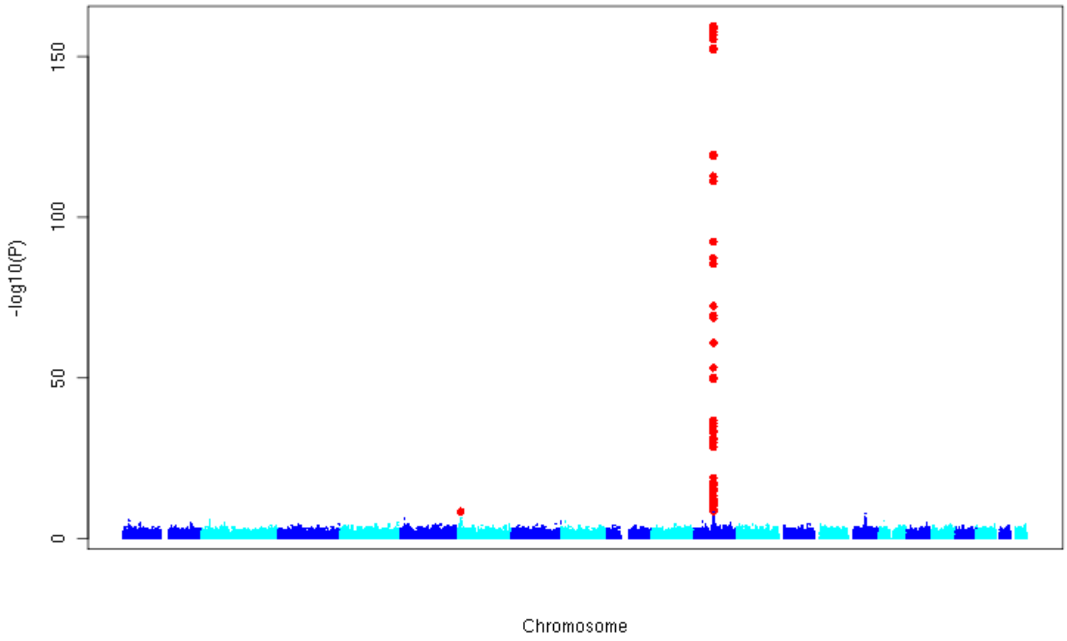

Metabolite Network 6

Composition of network (N = 4):

Metabolite abbreviation	Full description
bOHBuO	3-hydroxybutyrate
AcAcO	Acetoacetate
Cit	Citrate
Glol	Glycerol

Intra-correlation of network:

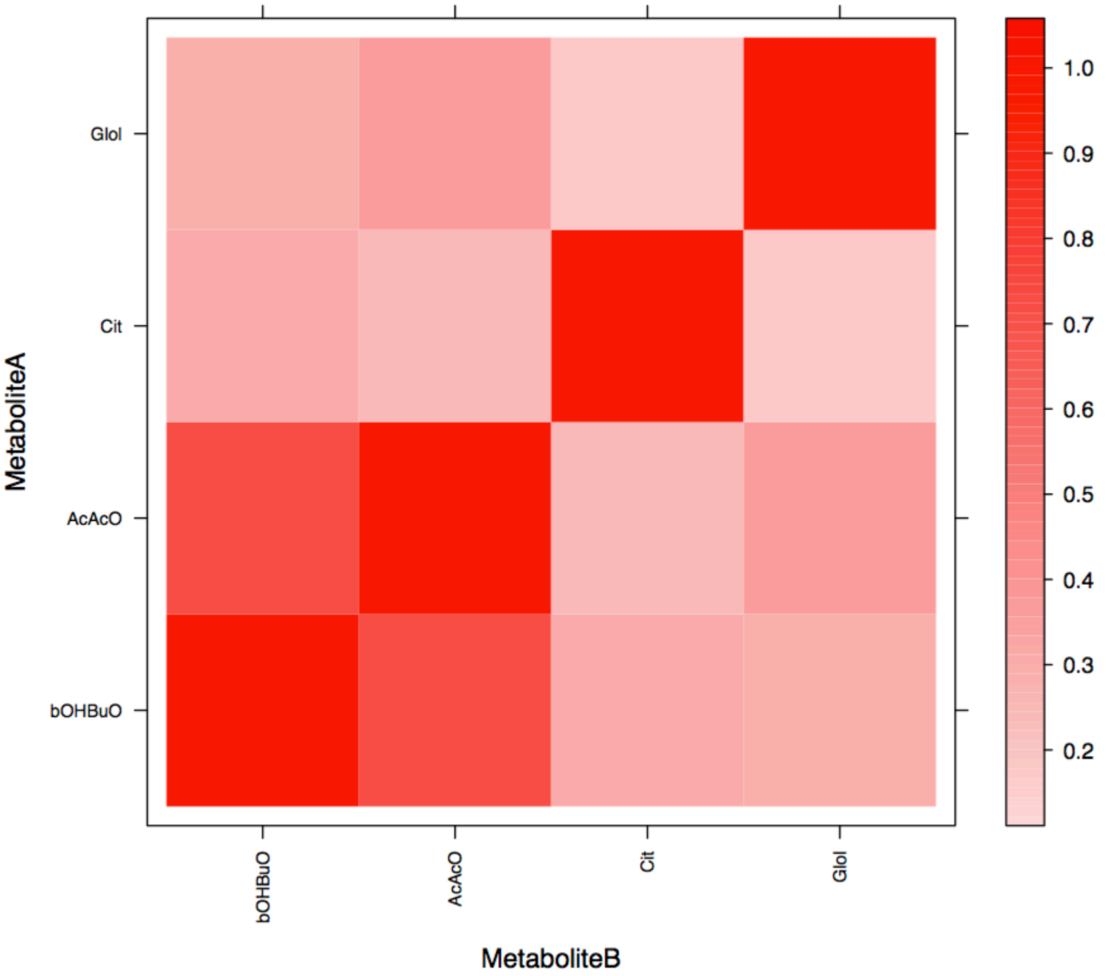

QQ plots:

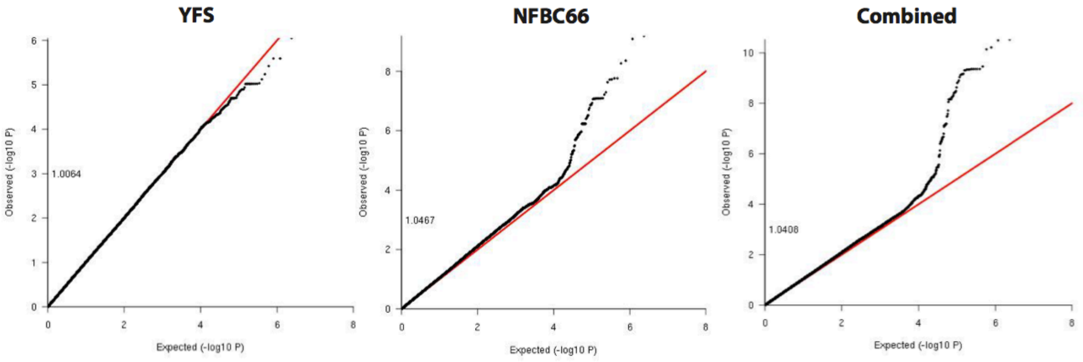

Comparison of multivariate and univariate *P* values:

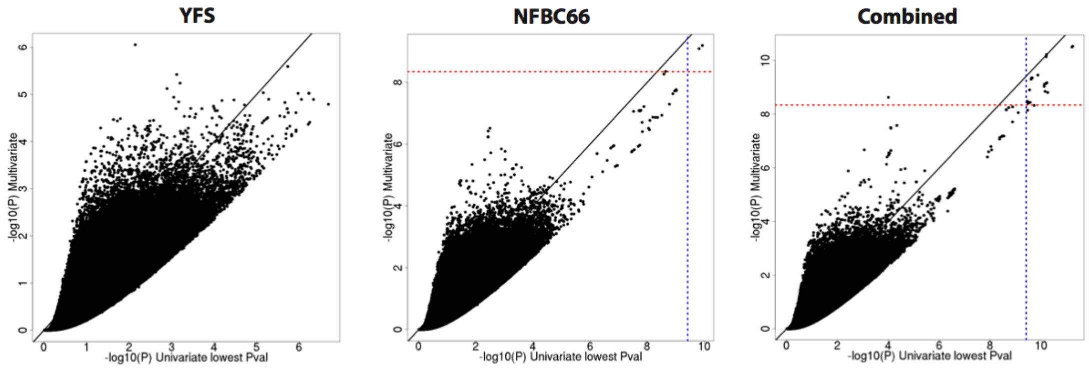

Genome-wide Manhattan plot for combined analysis:

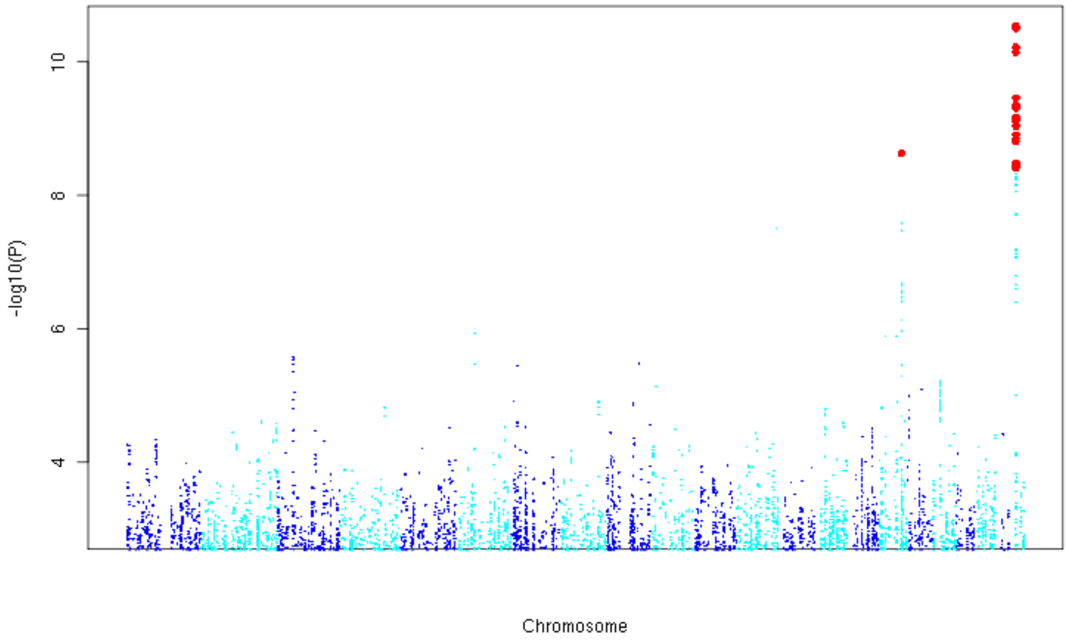

Metabolite Network 7

Composition of network (N = 3):

Metabolite abbreviation	Full description
Ala	Alanine
Lac	Lactate
Pyr	Pyruvate

Intra-correlation of network:

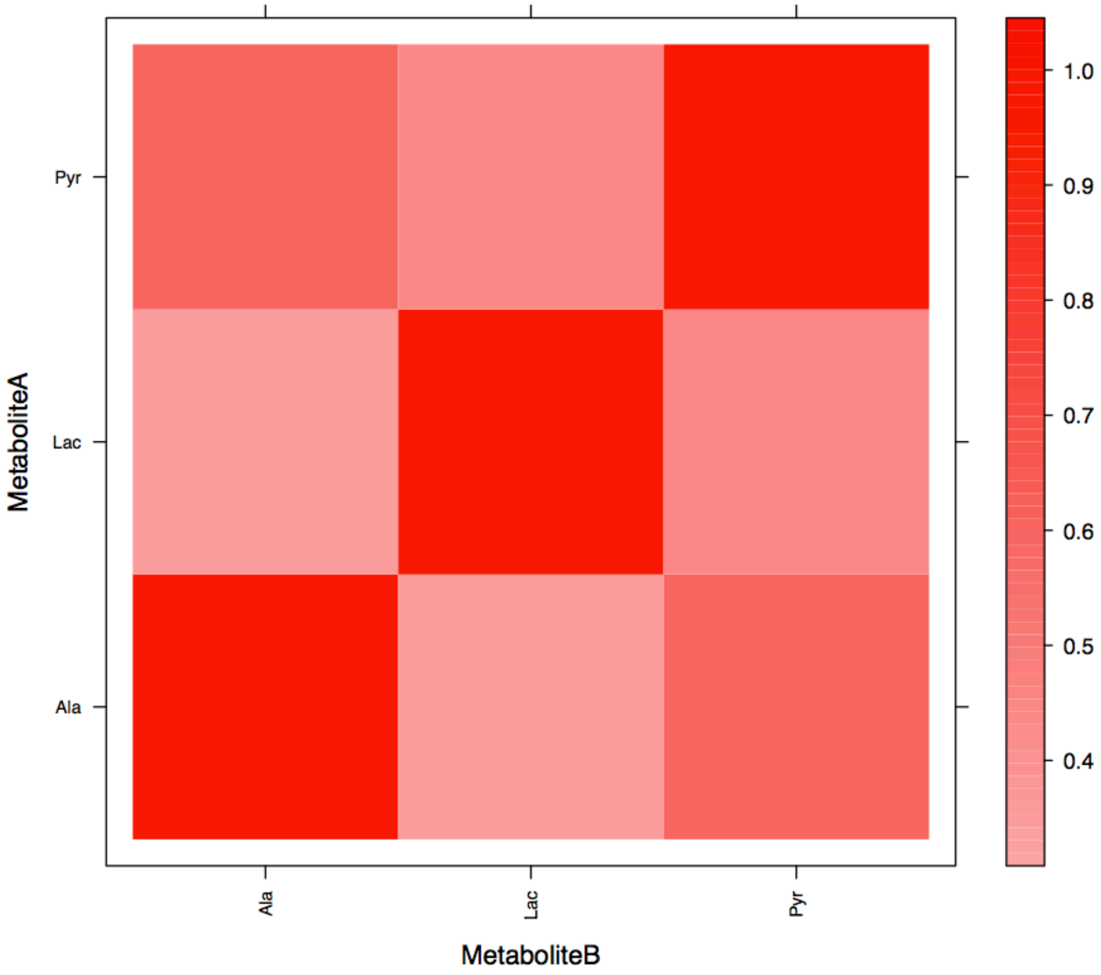

QQ plots:

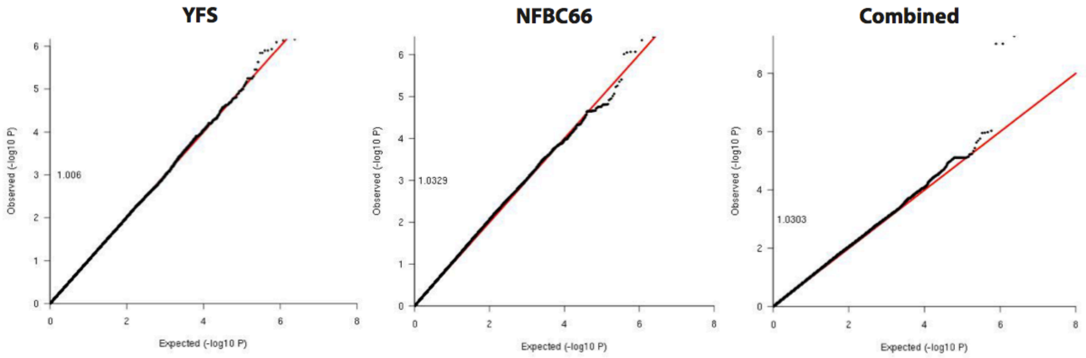

Comparison of multivariate and univariate *P* values:

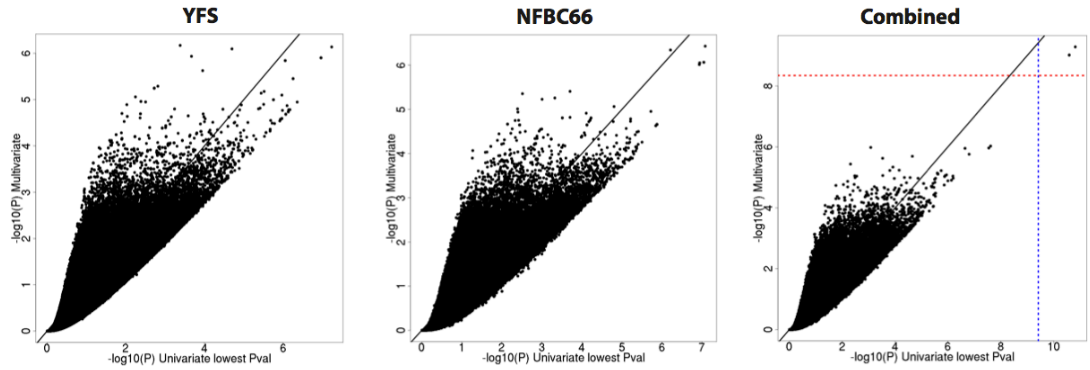

Genome-wide Manhattan plot for combined analysis:

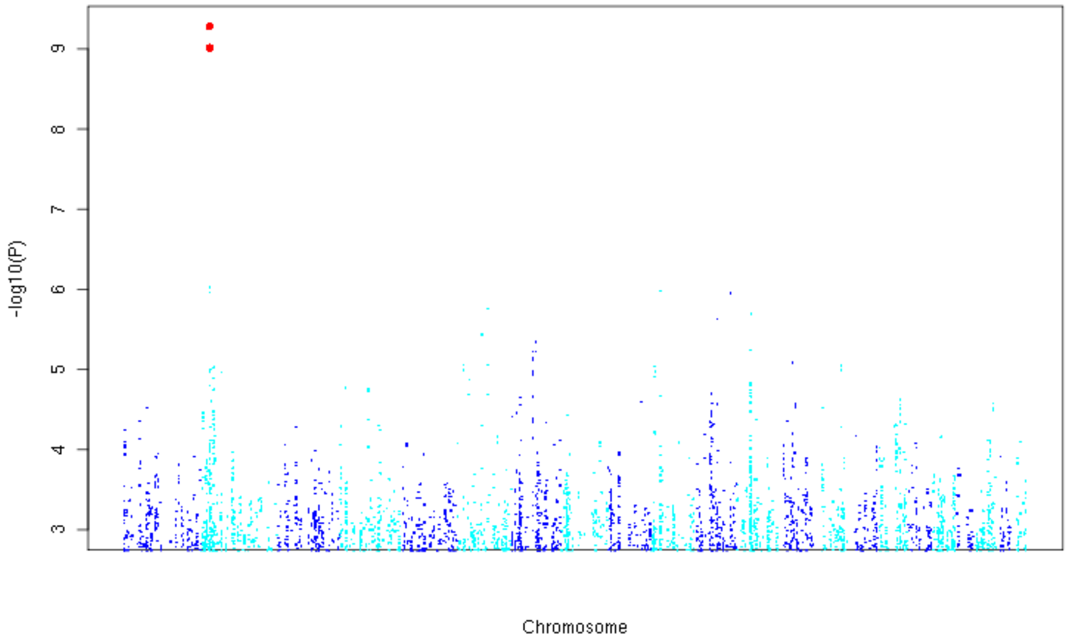

Metabolite Network 8

Composition of network (N = 3):

Metabolite abbreviation	Full description
Crea	Creatinine
Glc	Glucose
Gln	Glutamine

Intra-correlation of network:

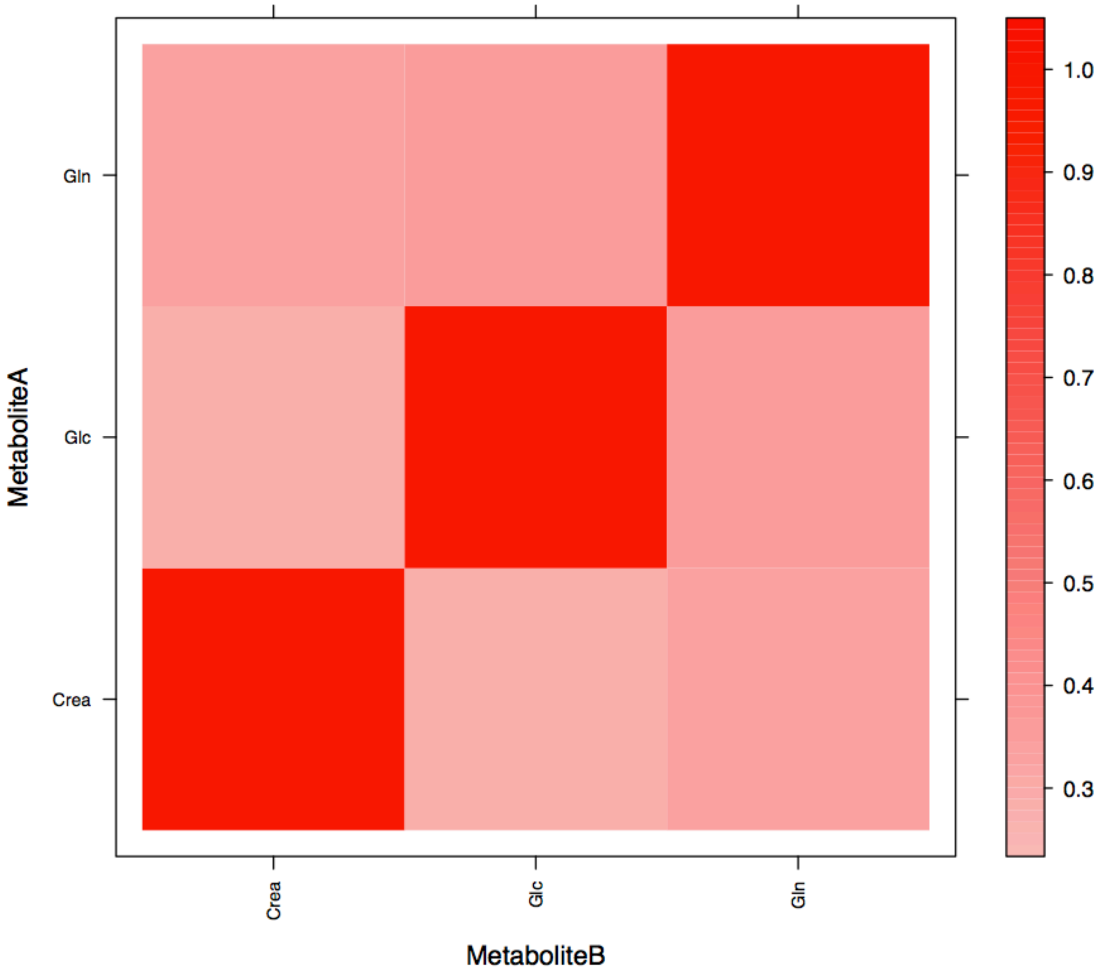

QQ plots:

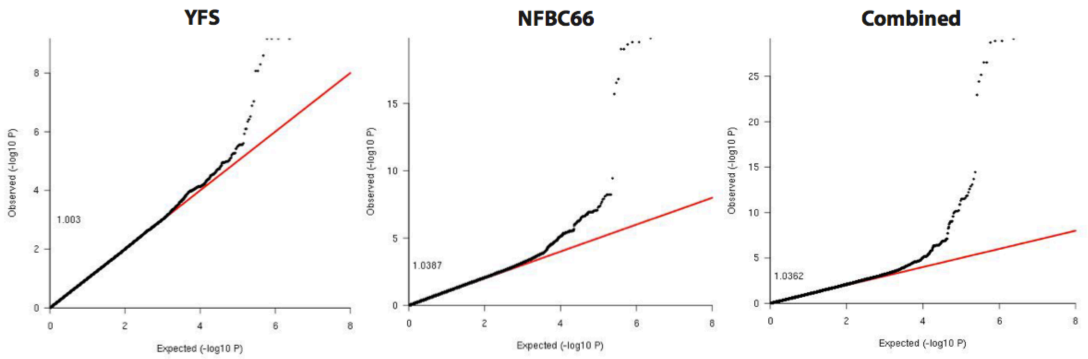

Comparison of multivariate and univariate *P* values:

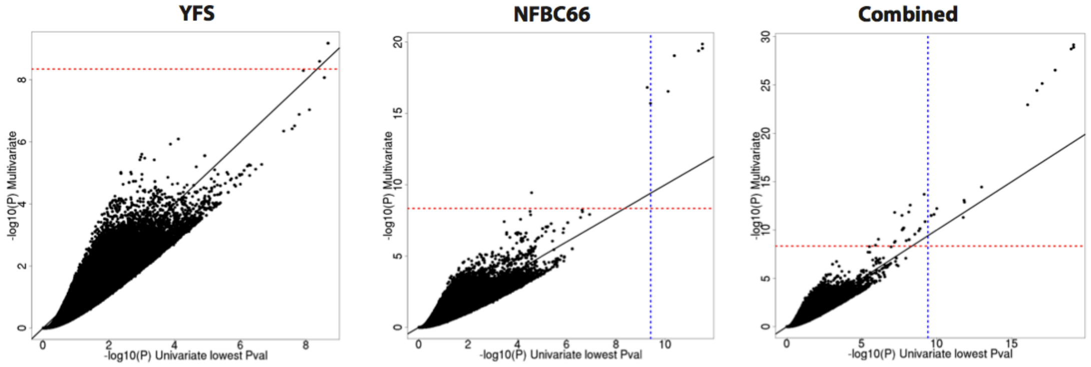

Genome-wide Manhattan plot for combined analysis:

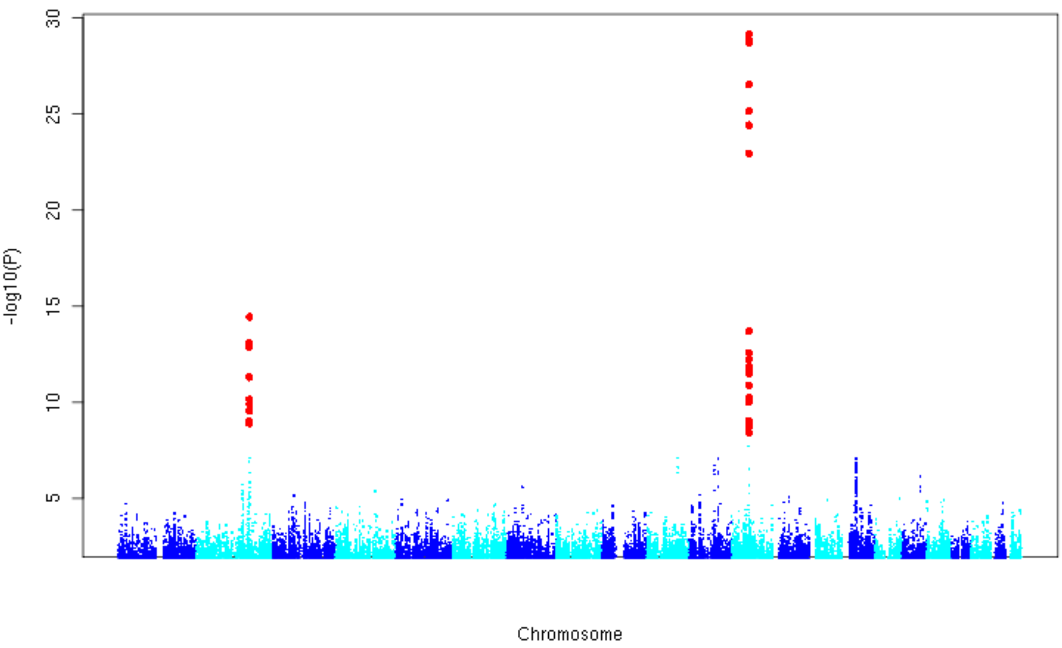

Metabolite Network 9

Composition of network (N = 2):

Metabolite abbreviation	Full description
CH2-FA	Average number of methylene groups in a fatty acid chain
FALen	Description of average fatty acid chain length, not actual carbon number

Intra-correlation of network:

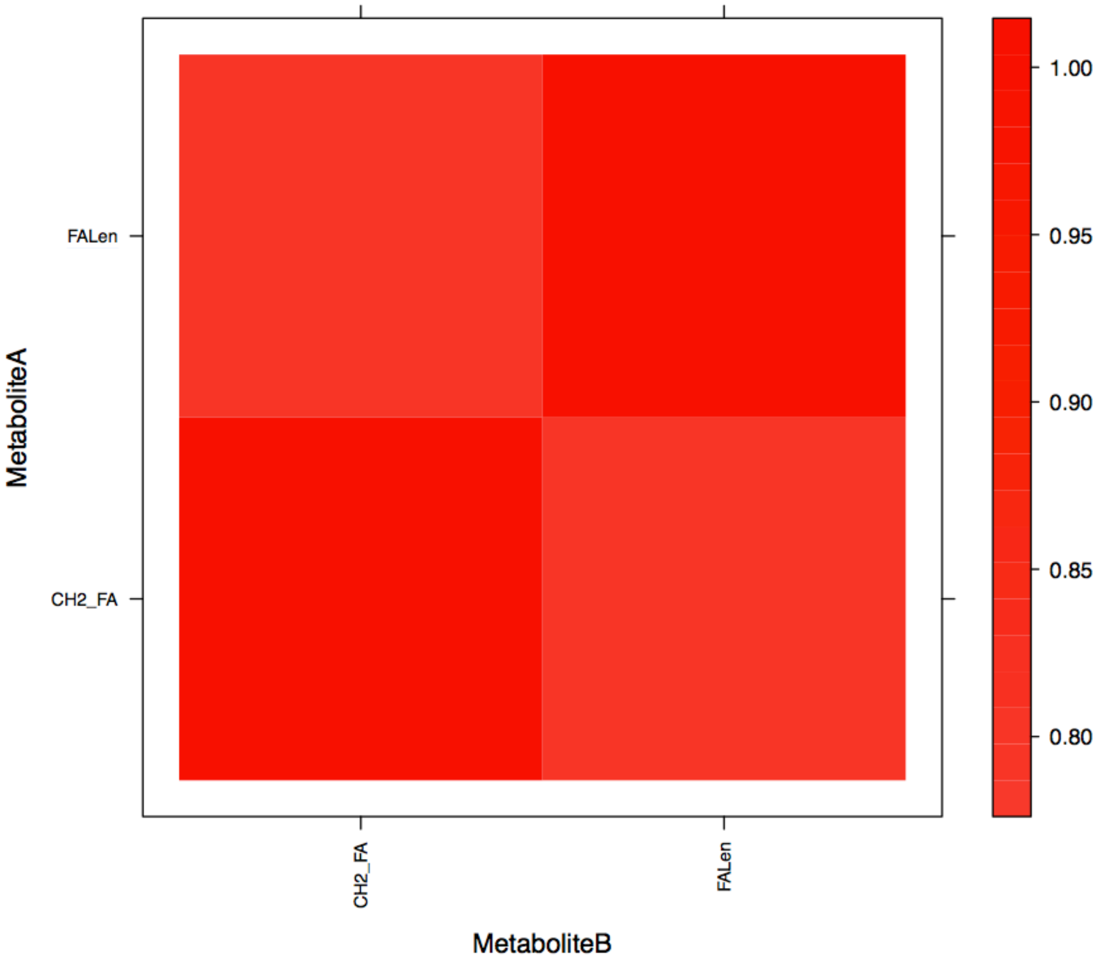

QQ plots:

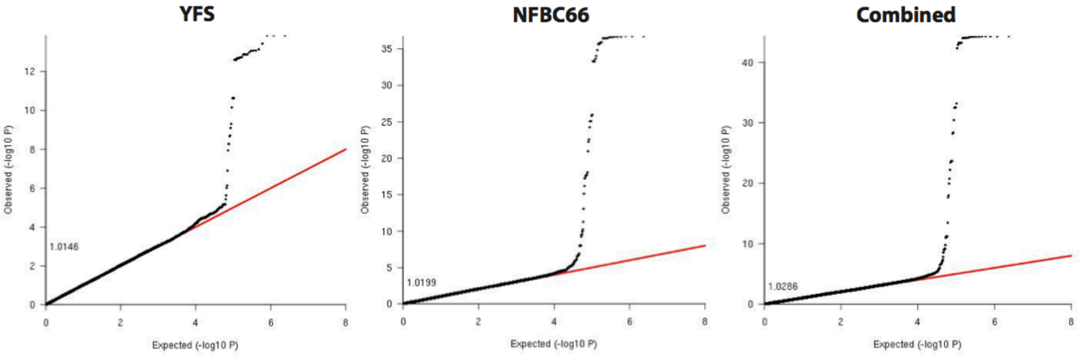

Comparison of multivariate and univariate *P* values:

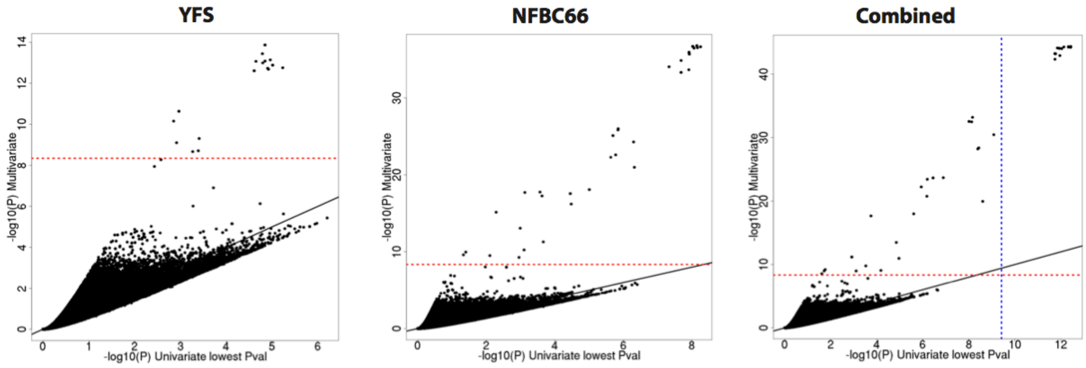

Genome-wide Manhattan plot for combined analysis:

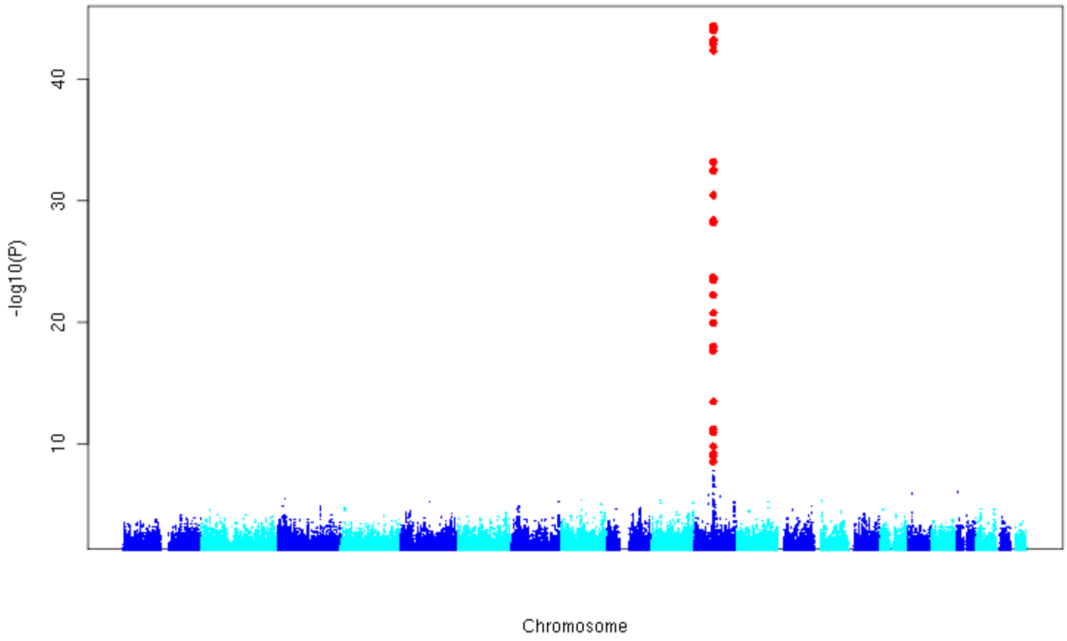

Metabolite Network 10

Composition of network (N = 2):

Metabolite abbreviation	Full description
LDL-D	Mean diameter for LDL particles
DB-FA	Average number of double bonds in a fatty acid chain

Intra-correlation of network:

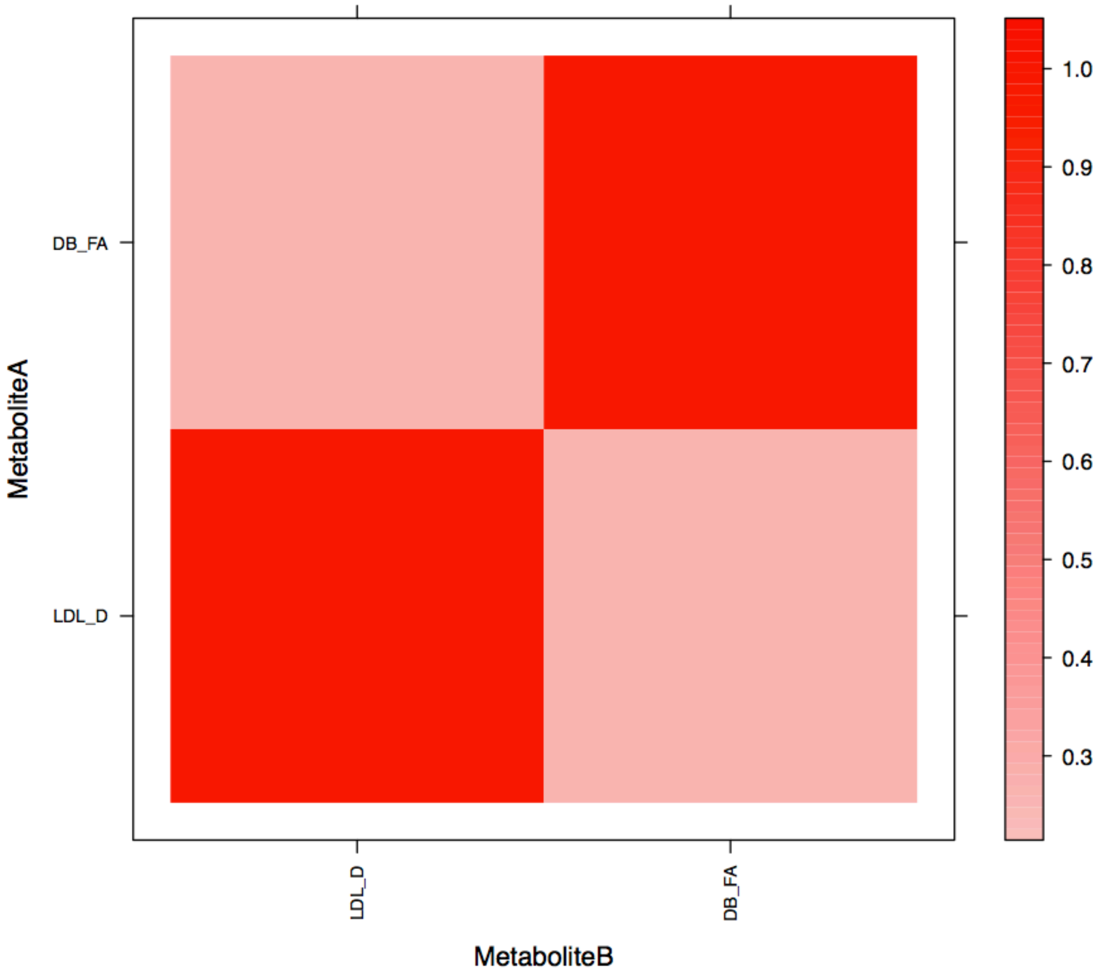

QQ plots:

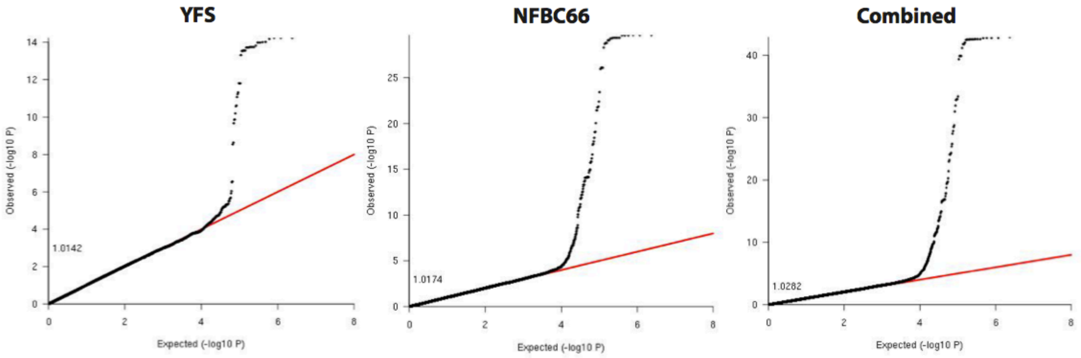

Comparison of multivariate and univariate *P* values:

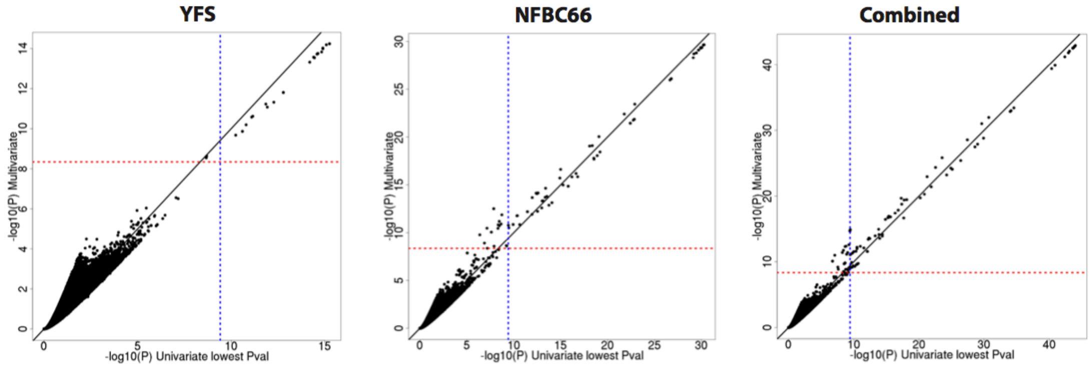

Genome-wide Manhattan plot for combined analysis:

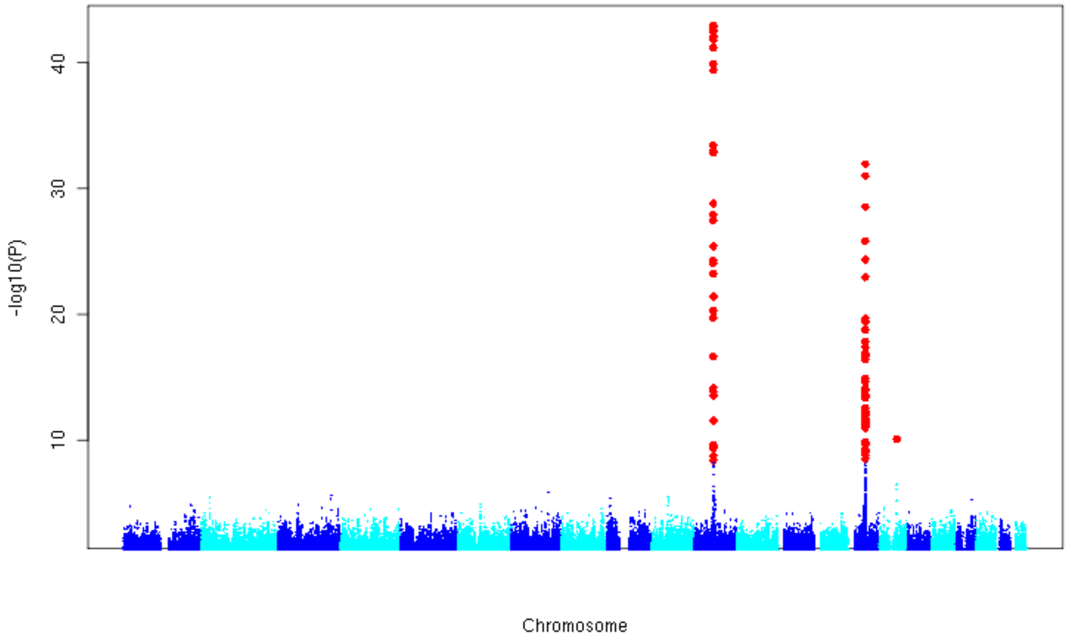

Metabolite Network 11

Composition of network (N = 2):

**Metabolite abbreviation**	**Full description**
AcO	Acetate
Urea	Urea

Intra-correlation of network:

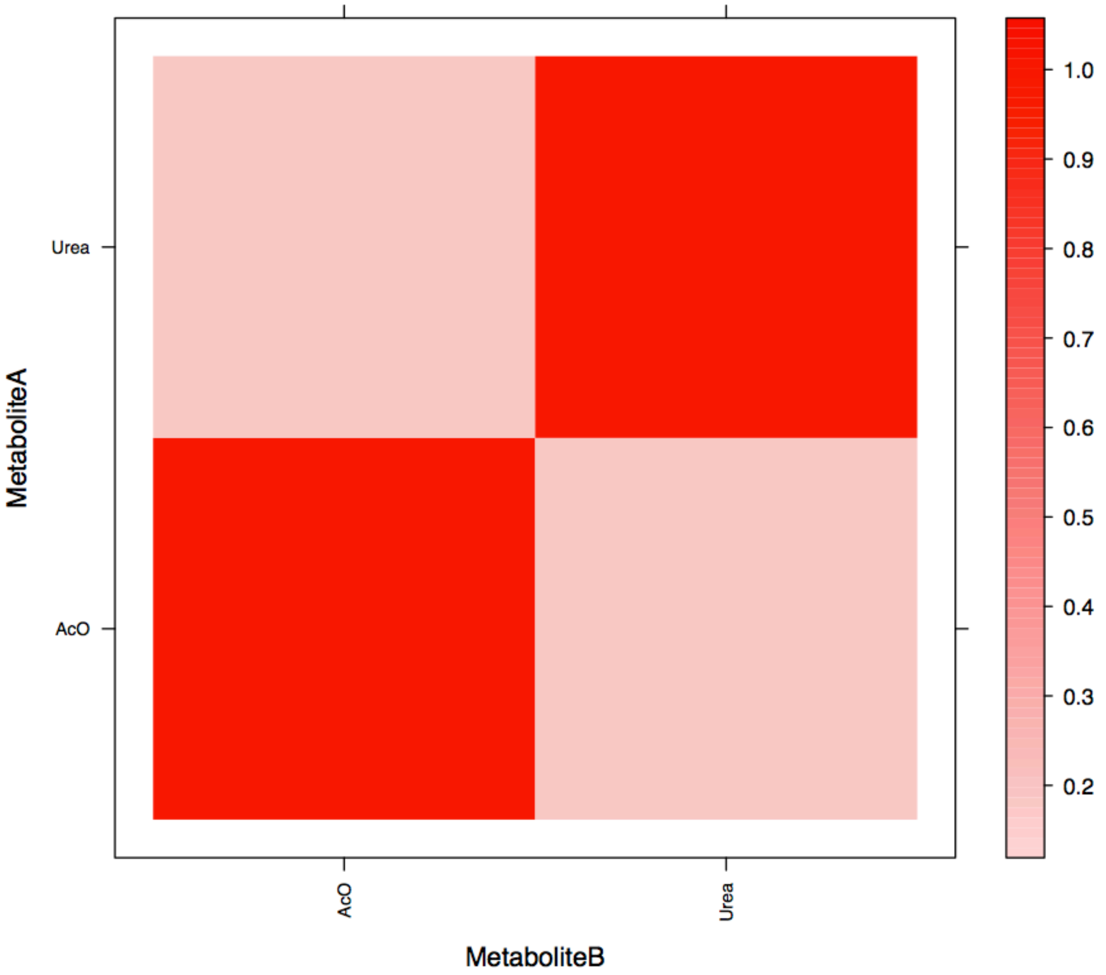

QQ plots:

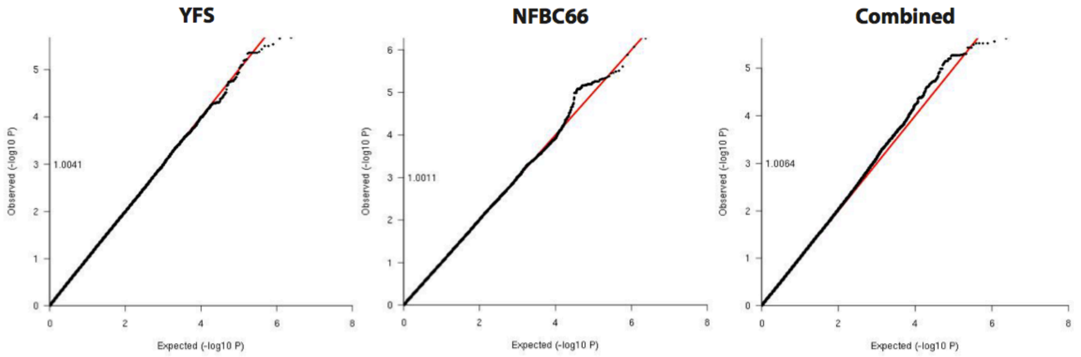

Comparison of multivariate and univariate *P* values:

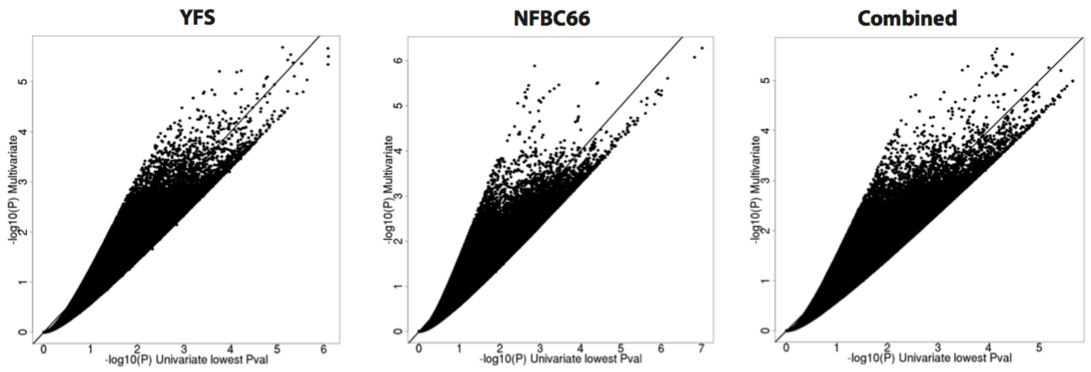

Genome-wide Manhattan plot for combined analysis:

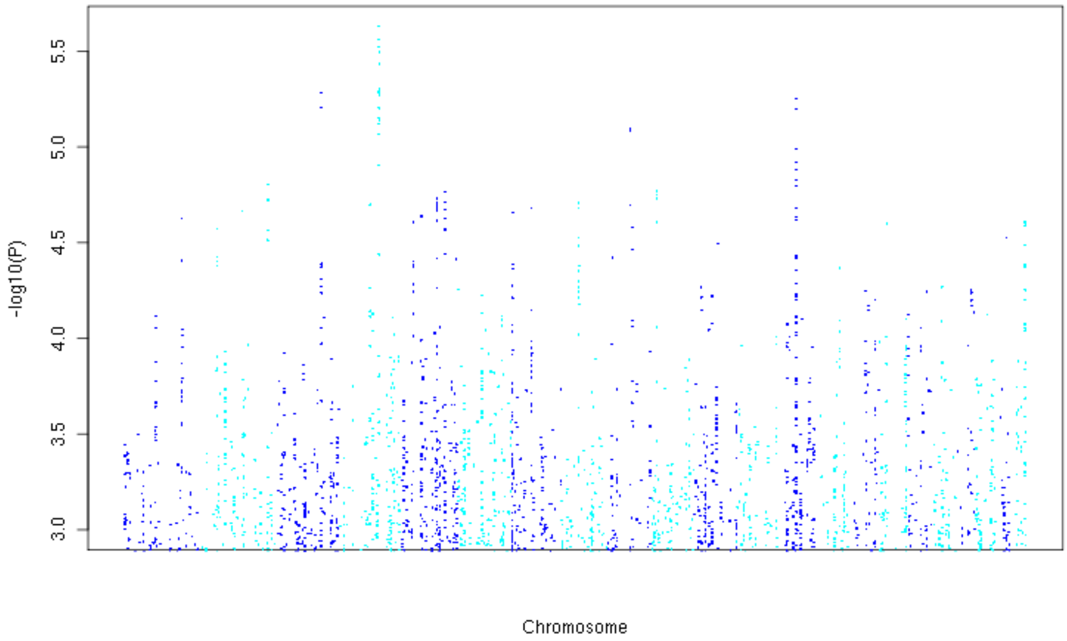

Supplement: Materials S1 — Section describing metabolic networks. Full descriptions, abbreviations, inter-metabolite correlations, and supporting association analyses. (PDF) [file pgen.1002907.s008.pdf]
